# Recovery and concordance in a secure forensic psychiatry hospital – the self rated DUNDRUM-3 programme completion and DUNDRUM-4 recovery scales

**DOI:** 10.1186/s12888-015-0433-x

**Published:** 2015-03-28

**Authors:** Mary Davoren, Sarah Hennessy, Catherine Conway, Seamus Marrinan, Pauline Gill, Harry G Kennedy

**Affiliations:** Department of Psychiatry, Trinity College Dublin, Dublin, Ireland; National Forensic Mental Health Service, Central Mental Hospital, Dundrum, Dublin 14, Ireland

## Abstract

**Background:**

Detention in a secure forensic psychiatric hospital may inhibit engagement and recovery. Having validated the clinician rated DUNDRUM-3 (programme completion) and DUNDRUM-4 (recovery) in a forensic hospital, we set out to draft and validate scales measuring the same programme completion and recovery items that patients could use to self-rate. Based on previous work, we hypothesised that self-rating scores might be predictors of objective progress including conditional discharge. We hypothesised also that the difference between patients’ and clinicians’ ratings of progress in treatment and other factors relevant to readiness for discharge (concordance) would diminish as patients neared discharge. We hypothesised also that this difference in matched scores would predict objective progress including conditional discharge.

**Method:**

In a prospective naturalistic observational cohort study in a forensic hospital, we examined whether scores on the self-rated DUNDRUM-3 programme completion and DUNDRUM-4 recovery scales or differences between clinician and patient ratings on the same scales (concordance) would predict moves between levels of therapeutic security and conditional discharge over the next twelve months.

**Results:**

Both scales stratified along the recovery pathway of the hospital, but clinician ratings matched the level of therapeutic security more accurately than self ratings. The clinician rated scales predicted moves to less secure units and to more secure units and predicted conditional discharge but the self-rated scores did not. The difference between clinician and self-rated scores (concordance) predicted positive and negative moves and conditional discharge, but this was not always an independent predictor as shown by regression analysis. In regression analysis the DUNDRUM-3 predicted moves to less secure places though the HCR-20 C & R score dominated the model. Moves back to more secure places were predicted by lack of concordance on the DUNDRUM-4. Conditional discharge was predicted predominantly by the DUNDRUM-3.

**Conclusions:**

Patients accurately self-rate relative to other patients however their absolute ratings were consistently lower (better) than clinicians’ ratings and were less accurate predictors of outcomes including conditional discharge. Quantifying concordance is a useful part of the recovery process and predicts outcomes but self-ratings are not accurate predictors.

**Electronic supplementary material:**

The online version of this article (doi:10.1186/s12888-015-0433-x) contains supplementary material, which is available to authorized users.

## Background

The recovery model for delivering mental health services has been adopted as policy in recent years by governments [1-4], regulators [5,6] and colleges [7]. Anthony defined recovery as a subjective, cognitive reframing - “A deeply personal unique process of changing one’s attitudes, values, feelings, goals, skills and/or roles” [8]. Resnick et al. described four key aspects to recovery: life satisfaction, hope and optimism, empowerment and knowledge about mental illness and services [9] – akin to a rights based empowerment. Davidson et al. regarded recovery as a process in which the person was “assuming control, managing symptoms and becoming empowered and exercising citizenship” [10]. The Sainsbury Centre for Mental Health combined these aspects, defining recovery as “about building a meaningful and satisfying life, as defined by the person themselves, whether or not there are ongoing or recurring symptoms or problems” [11]. These definitions of recovery focus on the importance of quality of life and may not necessarily include remission from symptoms. This view of recovery involves an individual taking long-term ownership of self-management, assuming both rights and responsibilities for managing his or her own health and avoiding relapse.

### Can recovery be implemented in a forensic mental health service?

In a recent position paper, the Sainsbury Centre for Mental Health highlighted the need to demonstrate use of the recovery model in forensic mental health services “Risk assessment and management need to become more open, more transparent with service users and staff working collaboratively together. This is particularly important in forensic and high risk settings, where recovery is just as important a principle as it is in any other part of the mental health service” [12]. Mezey et al. showed that the majority of in-patients in a secure forensic unit considered their involuntary admission to be a key part in their recovery [13]. In forensic mental health services most patients are detained under criminal law and mental health legislation and even when returned to the community, those who had been found not guilty by reason of insanity are usually conditionally discharged, with conditions intended to limit freedoms and restrict choices in order to minimise risk and protect the public. The challenge of making this process compatible with recovery was taken up by “the HCR-20 risk and recovery group” who involved patients in their own risk assessments and found that the programme offered their patients an improved understanding of the link between mental illness, risk and their detention [14]. However Troqete et al. combined risk assessment with shared care planning among a cohort of forensic out-patients and found that although case managers valued joint structured risk assessment with their patients, this joint approach did not reduce recidivism rates [15].

The therapeutic alliance between a patient and their multidisciplinary team is a key aspect of recovery, especially within forensic services, where many patients have a history of non-engagement with mental health services prior to offending [16]. Melzer et al. showed that non-compliance with treatment was one of the key factors leading to admission to 34 medium secure units in England and Wales [17]. Donnelly et al. showed that working alliance and interpersonal trust between in-patients and clinicians in a medium secure forensic hospital can be reliably measured and that ratings between in-patients and clinicians correlated [18]. Bressington et al. found that service users’ views of their therapeutic alliance with staff were strongly associated with satisfaction in secure mental health services [19]. Donnelly et al. also showed that positive symptoms, global function and measures of interpersonal trust and working alliance prior to a mental health tribunal hearing predicted satisfaction and perceived coercion with mental health hearings, irrespective of the outcome [20].

Implementing a recovery ethos is therefore shown to be possible in forensic mental health services, but practices and processes in forensic mental health services are assessed against hard outcomes such as length of stay as well as soft outcomes such as satisfaction [21].

### Can a recovery pathway be equated with a recovery process?

The recovery literature in forensic mental health appears to make a distinction between recovery pathways and recovery processes. The Central Mental Hospital is Ireland’s only secure forensic hospital and provides high, medium and low levels of therapeutic security integrated on one campus. On admission, patients are initially managed in high secure units then move onwards to medium secure units then low secure/pre-discharge units, which corresponds to a coherent pathway through secure care [22]. It has been shown that the placements according to levels of therapeutic security in a forensic mental health service correspond to measures of risk of harm to others and harm to self, symptom severity and global function in this [22-24] and other similar services [25]. Because each level of therapeutic security within the National Forensic Mental Health Service is linked to risk and needs assessments, this allows a clear and understandable connection between risk management and care planning, thereby providing patients with clarity and hope when working towards their own recovery [22]. Patient recovery is closely linked with engagement and progressive programme completion. It has often been thought that being detained in a secure hospital setting may be a barrier to patient engagement and true participation in therapeutic activities and programmes. We have therefore paid special attention to the engagement of patients in assessing treatment response and progress towards discharge [26].

The DUNDRUM Toolkit [26] Additional file 1 consists of five scales, the first two DUNDRUM-1 Triage Security [27] and DUNDRUM-2 Triage Urgency [28] are used for assisting decision making when admitting patients to a particular level of therapeutic security. The DUNDRUM-1 can also be used to benchmark case mixes (the average need for therapeutic security) when comparing study samples or the users of different services. The DUNDRUM-3 Programme Completion and DUNDRUM-4 Recovery scales [29] assist decision making when moving patients between levels of therapeutic security along the recovery pathway [30] or recommending patients for discharge to the community [31]. The DUNDRUM-3 programme completion items rate progress in relation to treatment programmes or ‘pillars of care’ including physical health, mental health, drugs and alcohol, problem behaviours, self-care and activities of daily living, education occupation and creativity, family and social networks. The DUNDRUM-4 recovery items include stability, insight, rapport, working alliance, leave, dynamic risk and victim sensitivities. We believe these items contain measures of both personal recovery and clinical recovery (as distinct from remission), since the barriers to recovery are social and contextual as well as personal. These have excellent psychometric properties [28-31] and these scales were associated with those patients who subsequently moved between levels of therapeutic security [29]. The DUNDRUM-1 was also a predictor of moves between levels of therapeutic security in the same hospital, along with a measure of risk, the HCR-20 dynamic score [30]. The DUNDRUM-3 and DUNDRUM-4 were the best predictors of conditional discharge to the community [31].

Having validated the DUNDRUM-3 and DUNDRUM-4 clinician rated measures of programme completion and recovery in the forensic hospital setting, we set out to draft and validate scales measuring the same programme completion and recovery items that patients could use themselves. This is the fifth scale, the Self Rated DUNDRUM Toolkit [32]. We were prompted by the service user led model of recovery firstly to model this self-rated needs assessment tool on the validated clinician rated structured professional judgement tools and secondly to develop it in collaboration with, rather than for service users in this forensic hospital setting. In the qualitative literature on recovery there appears to be no need to distinguish between subjective cognitive appraisals, rights-based policies, an ethos of recovery and an ethics of personal responsibility. In forensic mental health services there is an emphasis on quantitative research because of the need to demonstrate health gains using objectively measured outcomes such as discharge rates, relapse and reoffending rates. We concluded from an analysis of the literature that in forensic mental health services, the growth of agreement between clinicians and patients about issues such as completion of treatment programmes and other measures of progress along the recovery pathway would be a part of the process of recovery, as indicated by moves to less secure placements and conditional discharge.

We hypothesised that the self-rated scores for programme completion (DUNDRUM-3) and forensic recovery (DUNDRUM-4) would predict moves between levels of therapeutic security and conditional discharge with similar predictive accuracy to staff rated scores on the same scales. We also calculated the difference between pairs of clinician and patient ratings, as a measure of concordance. We hypothesised that this would represent a specific aspect of recovery in a forensic context, and increasing concordance (diminishing differences between staff and patient ratings) would also predict conditional discharge.

## Methods

### Design

This was a naturalistic prospective cohort study. The content of the self-rated versions was developed in consultation with a service user (SM) to allow ease of interpretation while ensuring fidelity to the clinician rated items.

Data were gathered as part of the clinical audit of service delivery. The study was approved by the National Forensic Mental Health Service research ethics, audit and effectiveness committee as a clinical audit project. Those who consented to participate agreed to allow their self-report form to be identifiable to the researchers though not to their treating clinicians, so that self-report and clinician rated reports could be collated. The clinician rated DUNDRUM-3 programme completion scale and DUNDRUM-4 recovery scale [32] were completed for all 97 patients in the Central Mental Hospital by MD in February 2012. The self-rated DUNDRUM-3 programme completion scale and DUNDRUM-4 recovery scales [32] were offered to all 97 patients by SH and CC, in February 2012 and completed by 64 patients (66%). SH and CC were blind to the ratings of MD. MD was not the decision maker for moves or for conditional discharge and the decision makers were blind to MD’s ratings. Treating clinicians, mental health review board members and MD were blind to the patients’ self-ratings.

Patients were observed for a fourteen month period after assessment, from November 2011 until December 2012. This period of observation did not overlap with the period of observation in the previous study of moves between levels of therapeutic security [30]. During the period of follow-up patients were observed for three binary outcomes. These were positive moves, i.e. the first move if any from a higher level of therapeutic security to a lower level, and also for negative moves i.e. the first move if any from a lower level of therapeutic security back to a higher level of security. Conditional discharge was decided by the Mental Health Review Board which was independent in the exercise of its statutory power to grant or withhold conditional or absolute discharge. All patients were reviewed by the Board at six monthly intervals.

### Variables: measurement instruments

In addition to the self-rated and clinician-rated DUNDRUM-3 and DUNDRUM-4, patients were rated for measures of risk of harm to self, using the Suicide Risk Assessment and Management Manual (S-RAMM) [33] and a measure of risk of harm to others, the Historical-Clinical-Risk-20 (HCR-20) [34] by the treating multidisciplinary teams and these were collated by MD.

### Statistical analysis

All data were entered into SPSS 20 [35] and confidence intervals for base rates were calculated with CIA [36]. Predictive utility was tested using the receiver operating characteristic (ROC) area under the curve (AUC). This is a composite of sensitivity and specificity. A significant result for the AUC is one that differs significantly from the ‘random’ AUC of 0.5 - as a minimum the lower limit of the 95% confidence interval for the AUC does not overlap 0.5. Correlation was measured using Spearman’s rank correlation coefficient.

Paired t-tests were used to compare clinician and self-rated scores on the DUNDRUM-3 and DUNDRUM-4 scales. Analysis of variance was used to compare those who went on to have positive moves with those who did not, and likewise for those who had negative moves and those who were granted conditional discharge.

Binary logistic regression was used to find the most parsimonious models for predicting positive moves, negative moves and conditional discharge. The Omnibus test of goodness of fit (X^2^), Cox and Snell R^2^ and Nagelkerke R^2^ tests were used as indicators of goodness of fit, with the Wald Χ^2^ statistic and odds ratio (Exp B) and 95% confidence interval of the odds ratio to indicate the effect of those factors remaining in the models generated.

## Results

A total of 97 patients were eligible for inclusion in the study, 89 male and 8 female patients. The mean age of the patients was 41 years (SD 12.3 years) and mean length of stay was 7.2 years (SD 9.7 years). The most common diagnoses according to ICD-10 criteria [37] were schizophrenia (ICD-10 F20) 71 (73%), schizoaffective disorder (ICD-10 F25) 8 (8%), bi-polar affective disorder (ICD-10 F31) 11 (11%), recurrent depressive disorder severe with psychotic symptoms (ICD-10 F33.3) 4 (4%) and intellectual disability (moderate mental retardation with significant impairment of behaviour ICD-10 F71.1) 3 (3%). The legal status was unfit to stand trial 8%, not guilty by reason of insanity 42%, prison to hospital transfer 34%, special transfer under the (civil) mental health 16%.

Of the 97 patients eligible at baseline, 64 (66%) completed the self-rated DUNDRUM-3 and DUNDRUM-4 instruments - 58 male and 6 female patients. No data were missing for participants. The mean DUNDRUM-1 triage security score was 29.9 (S.D.4.3) corresponding to a mean score per item for all eleven items of 2.7 (S.D. 0.4), and the mean score for the DUNDRUM-1 9 item scale (omitting suicide related items) was 2.9 SD 0.4) where a mean item score of ‘2’ would be typical of low security and ‘3’ would be typical of medium security, so that means of 2.7 and 2.9 are in keeping with a medium secure population. There was no difference in gender between those who participated versus those who declined (X^2^ = 0.316, p = 0.574). The mean follow up for those patients who participated was 402.5 days S.D. 127.7 and for those who declined 425.7 S.D. 99.7 (t = 0.93, df = 95, p = 0.364). When taking account of location at baseline [22,25,29,30], those who did and did not participate did not differ for clinician rated scores on the DUNDRUM-1 triage security scale (F = 0.512, df = 1, p = 0.477) DUNDRUM-3 (F = 3.325, df = 1, p = 0.072), DUNDRUM-4 (F = 3.558, df = 1, p = 0.063), HCR-20 total score (F = 2.471, df = 1, p = 0.120) or S-RAMM total score (F = 0.512, df = 1, p = 0.477).

**Figure 1:**
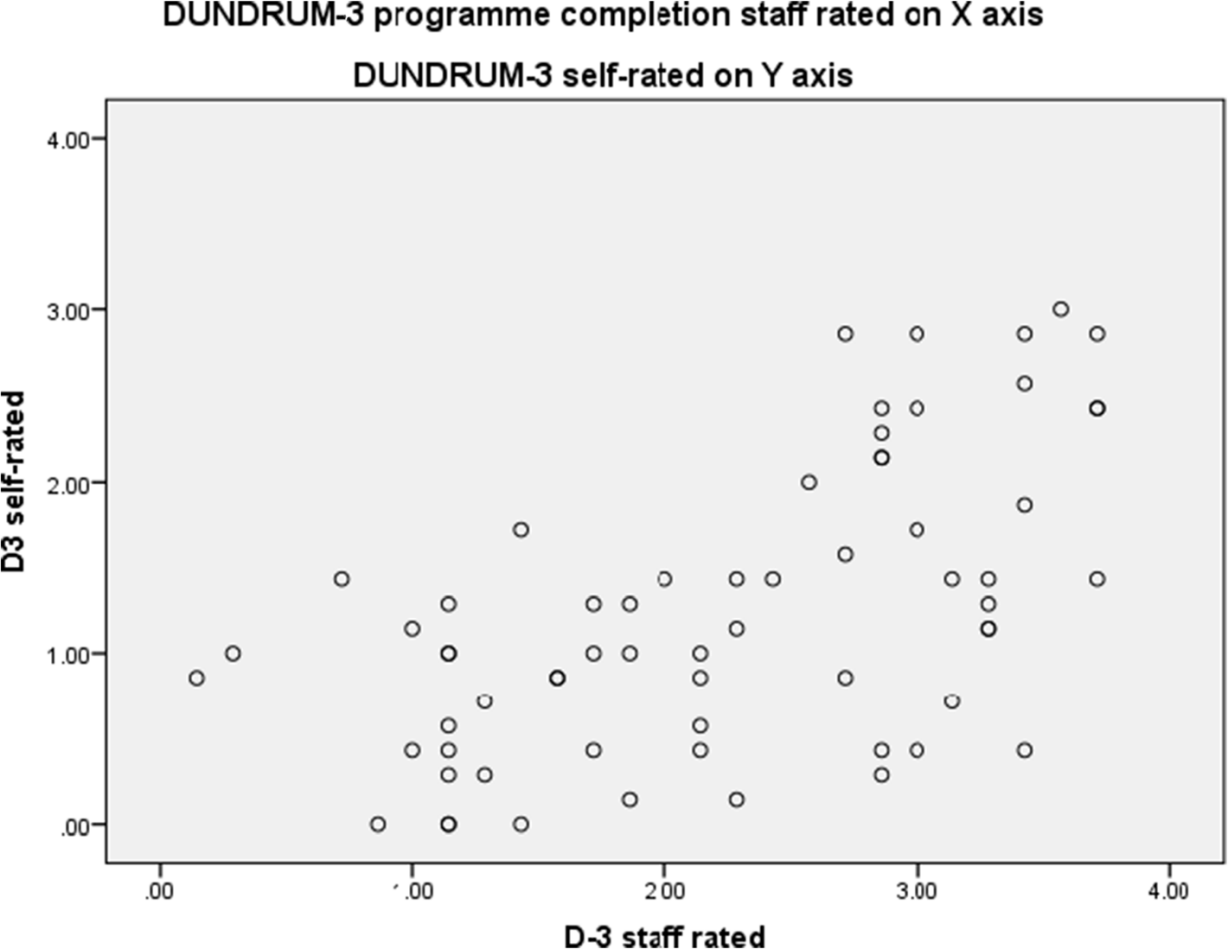
**DUNDRUM-3 programme completion staff rated v DUNDRUM-3 self-rated, Spearman r = 0.566, p < 0.001, n = 64.**

**Figure 2:**
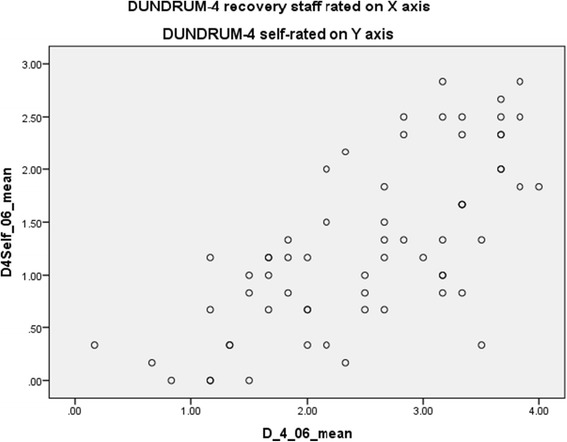
**DUNDRUM-4 recovery staff rated v DUNDRUM-4 self-rated, Spearman r = 0.712, p < 0.001, n = 64.**

Patients’ self-ratings on the DUNDRUM-3 and DUNDRUM-4 were significantly lower than staff ratings, showing that patients believed themselves to be further along their recovery pathway than clinicians did (Table 1). Figures 1 and 2 show that patients consistently rated themselves better than staff rated them, in relation to programme completion and forensic recovery scores. Table 1 shows that mean clinician rated scores for the DUNDRUM-3 and DUNDRUM-4, when divided by the number of items, correspond to the expected scores for each level of therapeutic security, with high secure patients averaging just above ‘3’, medium secure patients averaging just above ‘2’ and low secure patients averaging just above ‘1’. Self-rated mean scores however are consistently lower, with high secure patients rating themselves on average just above ‘2’ and medium secure patients rating themselves just above ‘1’. The differences are greatest for high and medium security, with diminishing differences for low secure and minimal secure or open placements.

**Table 1:** **The DUNDRUM-1 triage security scale was rated by admitting clinicians and has been divided by the number of items to normalise for the score 0–4, where greater than 3 indicates high security at the point of admission, 2 or more medium security, 1 or more low security and under 1 indicates minimal security needs**

		**DUNDRUM-1 triage security**		**DUNDRUM-3 programme completion**							**DUNDRUM-4 recovery**						
				**Clinician rated**		**Self-rated**		**Difference**		**Paired t test**	**Clinician rated**		**Self-rated**		**Difference**		**Paired t test**
	**n**	**Mean**	**S.D**	**Mean**	**S.D**	**Mean**	**S.D.**	**Mean**	**S.D.**	**t/df/p**	**Mean**	**S.D.**	**Mean**	**S.D.**	**Mean**	**S.D.**	**t/df/p**
High secure units	10	2.9	0.3	3.3	0.4	2.2	0.9	1.1	0.7	5.4/9/0.001	3.6	0.4	2.0	0.8	1.5	0.7	7.5/9/0.001
Medium secure units	25	2.9	0.4	2.7	0.6	1.4	0.6	1.3	0.9	7.3/24/0.001	2.9	0.6	1.6	0.6	1.3	0.7	9.5/24/0.001
Low secure units	11	2.7	0.3	1.6	0.4	0.7	0.4	0.9	0.7	4.5/10/0.001	1.9	0.6	0.7	0.4	1.2	0.6	6.4/10/0.001
Minimal/open units	12	2.9	0.4	1.0	0.5	0.6	0.5	0.4	0.8	1.7/11/0.111	1.4	0.6	0.5	0.5	0.9	0.6	4.8/11/0.001
Total	58	2.9	0.4	2.3	0.9	1.2	0.8	1.0	0.9	9.1/57/0.001	2.5	0.9	1.3	0.8	1.2	0.7	13.9/57/0.001

Clinician and self-rated scores for the DUNDRUM-3 programme completion and DUNDRUM-4 recovery scales, and the differences between them. DUNDRUM-3 scores have been divided by 7, the number of items in the scale, to normalise for the scoring 0–4, and DUNDRUM-4 scores have been divided by 6, the number of items in the scale for the same purpose.

### Internal consistency

The two clinician-rated instruments showed excellent internal consistency (DUNDRUM-3 Cronbach’s alpha = 0.904; DUNDRUM-4 Cronbach’s alpha = 0.881) as did the self-rated DUNDRUM-3 programme completion scale (Cronbach’s alpha = 0.844) and the self-rated DUNDRUM-4 recovery scale (Cronbach’s alpha = 0.731).

### Cross correlations

Cross correlations showed that the clinician rated DUNDRUM-3 and the self rated DUNDRUM-3 correlated (Spearman correlation coefficient r = 0.566, p < 0.001) and the clinician-rated DUNDRUM-4 recovery scale and the self-rated DUNDRUM-4 correlated with each other (r = 0.712, p < 0.001) (Figures 1 and 2).

### Moves between levels of therapeutic security

The 64 patients who completed the self rated scale consisted of 58 male patients and six female patients. In the Central Mental Hospital, female patients have a different recovery pathway within the hospital and so were not considered eligible for moves between levels of security. Of the 58 male patients who participated in the self-rated assessment and were eligible for moves between levels of therapeutic security, 27 had positive moves, 8 had negative moves and 23 had no moves during the 14 month follow-up period. Total follow up time for those patients who participated was 23,385.3 days. This yielded a base rate of 421.71 positive moves per 1,000 patient years (95% confidence interval 277.9-613.6) and a rate of 124.9 negative moves per 1,000 person years (95% confidence interval 53.9-246.2) [38]. Of the remaining 31 patients who declined to participate but were eligible for moves between levels of therapeutic security, 10 patients had positive moves, 3 had negative moves and 18 had no move. Overall there was no significant difference in the number of moves between levels of therapeutic security among those patients who participated versus those patients who declined to participate, with no difference between the two groups in positive moves (X^2^ = 0.726, df = 1, p = 0.394) or negative moves (X^2^ = 0.316, df = 1, p = 0.574).

In this observation period, location at baseline did not predict positive moves, with receiver operating characteristic area under the curve (AUC) = 0.585 (95% CI 0.436-0.734, p = 0.267) or negative moves (AUC = 0.614, 95% CI 0.415-0.812, p = 0.305) though it did predict conditional discharge for those eligible (AUC = 0.912, 95% CI 0.827-0.996, p < 0.001). As before, the HCR-20 dynamic (C+R) score predicted positive moves (AUC = 0.791, 95% CI 0.675-0.908, p < 0.001) negative moves (AUC = 0.706, 95% CI 0.550-0.863, p = 0.063) and conditional discharge (AUC = 0.865, 95% CI 0.740-0.990, p = 0.004). The DUNDRUM-1 triage security score did not predict positive or negative moves and as before did not predict conditional discharge.

### Positive moves

Table 2 shows that those who went on to have positive moves from higher levels of therapeutic security to lower levels, had lower scores on the clinician rated DUNDRUM-3 programme completion scale (AUC = 0.718, 95% CI 0.586-0.849, p = 0.005). The clinician rated DUNDRUM-4 recovery scale also predicted those patients who went on to have positive moves (AUC = 0.695, 95% CI 0.556-0.833, p = 0.011). However the self rated DUNDRUM-3 programme completion scale did not predict those patients who went on to have positive moves (AUC = 0.568, 95% CI 0.419-0.717, p = 0.377). Neither did the self rated DUNDRUM-4 recovery scale (AUC = 0.576, 95% CI 0.428-0.725, p = 0.321).

**Table 2:** **The clinician rated and self-rated DUNDRUM-3 and DUNDRUM-4, and the differences between the two, as predictors of positive moves, negative moves and conditional discharges**

	**No**		**Yes**		**ANOVA**	**AUC**	**95% CI**		**p**
	**Mean**	**S.D.**	**Mean**	**S.D.**	**F/p**		**Lower**	**Upper**	
	Positive moves								
DUNDRUM-3									
N	32		26						
Clinician rated	2.6	0.9	1.9	0.9	9.4/0.003	0.718	0.586	0.849	0.005
Self rated	1.4	0.9	1.1	0.7	1.4/0.240	0.508	0.419	0.717	0.377
Difference	1.2	0.7	0.8	0.9	4.7/0.035	0.640	0.493	0.787	0.069
DUNDRUM-4									
Clinician rated	2.8	0.9	2.2	0.9	6.7/0.012	0.695	0.556	0.833	0.011
Self rated	1.4	0.9	1.2	0.7	1.1/0.304	0.576	0.428	0.725	0.321
Difference	1.4	0.6	1.0	0.8	5.6/0.022	0.688	0.544	0.828	0.015
	Negative moves								
DUNDRUM-3									
N	50		8						
Clinician rated	2.1	0.8	2.9	0.8	5.4/0.023	0.760	0.588	0.932	0.019
Self rated	1.2	0.8	1.4	0.8	0.3/0.616	0.586	0.383	0.790	0.437
Difference	0.9	0.9	1.6	0.4	4.4/0.140	0.776	0.643	0.890	0.013
DUNDRUM-4									
Clinician rated	2.4	0.9	3.3	0.6	6.9/0.011	0.784	0.640	0.927	0.010
Self rated	1.3	0.8	1.4	0.7	0.3/0.613	0.552	0.354	0.751	0.636
Difference	1.1	0.7	1.9	0.4	9.8/0.003	0.840	0.727	0.953	0.002
	Conditional discharge								
DUNDRUM-3									
N	52		6						
Clinician rated	2.4	0.9	0.8	0.3	14.3/0.001	0.961	0.911	0.999	0.001
Self rated	1.3	0.8	0.9	0.5	1.1/0.299	0.624	0.426	0.821	0.361
Difference	1.1	0.8	−0.0	0.7	9.6/0.003	0.851	0.690	0.999	0.010
DUNDRUM-4									
Clinician rated	2.6	0.9	1.5	0.3	6.4/0.014	0.844	0.742	0.946	0.011
Self rated	1.3	0.8	0.8	0.4	1.7/0.193	0.678	0.526	0.830	0.189
Difference	1.3	0.7	0.7	0.2	3.4/0.070	0.771	0.651	0.891	0.045

DUNDRUM-3 scores have been divided by 7, the number of items in the scale, to normalise for the scoring 0–4, and DUNDRUM-4 scores have been divided by 6, the number of items in the scale for the same purpose.

### Negative moves

Those who went on to have negative moves, from lower levels of therapeutic security back to higher levels had significantly higher scores on the clinician rated DUNDRUM-3 programme completion scale (AUC = 0.760, 95% CI 0.588-0.932, p = 0.019). The clinician rated DUNDRUM-4 recovery scale also predicted those patients who went on to have negative moves (AUC = 0.784, 95% CI 0.640-0.927, p = 0.010) (Table 2). However the self rated DUNDRUM-3 programme completion scale did not predict those patients who went on to have negative moves (AUC = 0.586, 95% CI 0.383-0.790, p = 0.437). Neither did the self-rated DUNDRUM-4 recovery scale (AUC = 0.552, 95% CI 0.354-0.751, p = 0.636).

### Conditional discharge from the forensic hospital setting

Among the patient group studied, only those patients who had been found Not Guilty by Reason of Insanity (NGRI) or Unfit to Stand Trial, were eligible for conditional discharge from the forensic hospital, under Irish Law. Of the 64 patients who completed the self rated scale, 58 were eligible for conditional discharge (not the same as the 58 males who participated). There were 6 conditional discharges in this follow up period, a base rate of 6/25,544 days or 6/69.936 years, or 85.8 per 1,000 patient years (95% confidence interval 31.5 to 186.8 per 1,000 patient years). All six patients who went on to receive a conditional discharge had participated in the self-rating study.

Table 2 shows that the clinician rated DUNDRUM-3 programme completion scale predicted those patients who went on to be granted conditional discharge to the community (AUC = 0.961, 95% CI 0.911-0.999, p < 0.001), as did the clinician rated DUNDRUM-4 recovery scale (AUC = 0.844, 95% CI 0.742-0.946, p = 0.011). The self rated DUNDRUM-3 programme completion scale did not predict conditional discharge to the community (AUC = 0.624, 95% CI 0.426-0.821, p = 0.361), nor did the self rated DUNDRUM-4 recovery scale (AUC = 0.678, 95% CI 0.526-0.830, p = 0.189).

### Concordance

Table 1 also shows that not only the absolute scores, but the differences between clinician and self-rated scores were lower (concordance was higher) for those patients who had progressed to the minimal security/pre-discharge units of the hospital. The progressive decline in the difference between clinician and self-rated DUNDRUM-3 programme completion scores from high secure through medium to low secure and minimal secure open units was significant for linear trend (linear by linear Χ^2^ = 6.1, df = 1, p = 0.014) and for DUNDRUM-4 recovery score differences between clinician and self-rated scores (linear by linear Χ^2^ = 5.9, df = 1, p = 0.015).

Table 2 shows that the difference between clinician and self-rated scores was significantly less (concordance was better) for those who had positive moves, the difference was greater (concordance was less good) for those who had negative moves and the difference was least (concordance was best) for those who were granted conditional discharge by the Mental Health Review Board.

This measure of increasing concordance may be a marker of patient recovery in so far as need for lesser levels of therapeutic security may be equated with recovery in a forensic setting.

### Secondary analysis

Binary logistic regression was used to examine the extent to which the variables associated with positive and negative moves and conditional discharge were independent of other identified predictors. Table 3 shows the results for forward entry likelihood ratio models. Each model included the clinician rated DUNDRUM3 and DUNDRUM-4, the patient self-rated DUNDRUM-3 and DUNDRUM-4, and the differences between the paired clinician and patient ratings for DUNDRUM-3 and for DUNDRUM-4. When the model for the first six variables had been derived for each of the three outcome measures, the models were repeated this time adding the HCR-20 dynamic (C + R) scores.

**Table 3:** **Binary logistic regression modelling**

	**Omnibus test df = 1**		**Cox & Snell**	**Nagelkerke**	**Correctly classified**	**B**	**SE**	**Wald df = 1**		**Odds ratio/Exp (B)**	**95% CI of odds ratio**	
	**X** ^**2**^	**p**	**R** ^**2**^	**R** ^**2**^	**%**			**X** ^**2**^	**p**		**Lower**	**Upper**
**Outcome: Positive moves**												
MODEL 1	8.7	0.003	0.139	0.187	65.5							
DUNDRUM-3 clinician rated						−0.124	0.045	7.55	0.006	0.884	0.809	0.965
Constant						1.725	0.753	5.25	0.022	5.614		
MODEL 2, add HCR-20 (C + R)	15.7	0.001	0.237	0.317	75.9							
HCR-20 (C + R)						−0.264	0.078	11.33	0.001	0.768	0.659	0.896
Constant						1.423	0.543	6.87	0.009	4.149		
**Outcome: Negative moves**												
MODEL 3	9.06	0.003	0.145	0.262	86.2							
DUNDRUM-4 clinician-patient difference						0.319	0.125	6.54	0.011	1.375	1.077	1.756
Constant						−4.730	1.347	12.33	0.001	0.009		
MODEL 4, add HCR-20 (C + R)	9.06	0.003	0.145	0.262	86.2							
DUNDRUM-4 clinician-patient difference						0.319	0.125	6.54	0.011	1.375	1.077	1.756
Constant						−4.730	1.347	12.33	0.001	0.009		
**Outcome: Conditional discharge**												
MODEL 5	26.2	0.001	0.336	0.604	95.3							
DUNDRUM-3 clinician rated						−0.553	0.214	6.69	0.010	0.575	0.378	0.875
Constant						3.965	1.864	4.53	0.033	52.737		
MODEL 6 add HCR-20 (C + R)	26.2	0.001	0.336	0.604	95.3							
DUNDRUM-3 clinician rated						−0.553	0.214	6.69	0.010	0.575	0.378	0.875
Constant						3.965	1.864	4.53	0.033	52.737		

All models include clinician rated DUNDRUM-3 and DUNDRUM-4, patient self-rated DUNDRUM-3 and DUNDRUM-4 and the differences between pairs. Models 2, 4 and 6 also include the HCR-20 (C + R) dynamic score. All models: forward stepwise likelihood ratio.

For positive moves, model 1 had a satisfactory fit and only the clinician rated DUNDRUM-3 remained in the model as a predictor of positive moves. The higher the DUNDRUM-3 clinician rated score (the less progress a patient had made in treatment programmes) the less likely the patient was to move to a less secure unit. Adding the HCR-20 dynamic score to the first six variables (model 2) dominated the model, with only the HCR-20 dynamic score remaining in the model as a predictor.

For negative moves, model 3 showed that the difference between the clinician rated DUNDRUM-4 recovery score and the patient self-rated DUNDRUM-4 recovery score was the only variable that remained in the model. The greater the difference between the two scores (the less concordance between clinician and patient) the more likely the patient was to be moved back from a lower to a more secure unit. Adding the HCR-20 dynamic score to the first six variables (model 4) made no difference.

For conditional discharge, model 5 shows that the DUNDRUM-3 clinician rated programme completion score was the only remaining predictor in the model. The greater the progress in treatment as rated by clinicians, the more likely the patient was to be conditionally discharged. Adding the HCR-20 dynamic score to the first six variables made no difference to the model.

The addition of the DUNDRUM-1 triage security score made no difference to any of these models.

## Discussion

### Main findings

In this study the self rated and clinician rated measures of programme completion and recovery correlated well. However the patients rated themselves more optimistically than the clinicians did and they rated themselves further along in their recovery process than their current placement would indicate. Using the DUNDRUM-3 programme completion and DUNDRUM-4 recovery scales we found that patients accurately rated themselves relative to other patients, however their absolute ratings appeared to lack precision. The patient ratings did not predict moves between levels of therapeutic security or conditional discharge. This is not simply a difference of opinion. The clinician ratings were significant predictors of conditional discharge, a decision made by a legally constituted Mental Health Review Board that was by statute independent in the exercise of its powers. Concordance, a measure of agreement between the clinician and patient ratings, improved from high to medium to low secure and minimal secure groups, with increasing concordance (diminishing differences) signaling the likelihood of discharge. This measure of concordance was a statistically significant predictor of moves and conditional discharge although it was not an independent predictor of positive moves or conditional discharge, as indicated by binary logistic regression.

We had previously shown that patient and clinician ratings regarding placement were less well correlated, with the same tendency for patients to rate themselves more optimistically than their clinicians [38]. Using the Camberwell Assessment of Need, Forensic Version (CANFOR), [39] we found in another study that patient ratings of their own unmet needs were consistently lower (less problematic) than staff ratings of the unmet needs of the same patients [22]. We have also shown that patients and clinicians correlated well in their ratings of therapeutic rapport and interpersonal trust, again with patients rating this more positively than the clinician ratings [18]. These findings are in keeping with similar findings in other settings. Killaspy et al. found that when rating the mental health recovery star, collaborative ratings between staff and patients were higher (better) than ratings completed by staff alone [40]. The lack of precision and lack of predictive accuracy shown by patient self ratings may be the same phenomenon underlying the failure of joint rating and shared care planning in Troqete et al.’s study using the HCR-20 [15].

This study replicates our earlier studies concerning clinician ratings of the DUNDRUM-3 and DUNDRUM-4 for an overlapping sample of patients but for a different time period. This period of observation had a much greater number of positive moves because of the introduction of legislation permitting conditional discharge. In this study the clinician-rated DUNDRUM-3 and DUNDRUM-4 scores were significantly better (lower) for positive moves and significantly higher (worse) for negative moves while lower (better) scores also predicted conditional discharge. The earlier study showed that the DUNDRUM-1 triage security scale, a measure of dangerousness and the HCR-20 ‘dynamic’ measure of risk along with location at baseline, were stronger predictors of positive and negative moves [30], though the clinician rated DUNDRUM-3 and DUNDRUM-4 were the best predictors of conditional discharge [31]. This study, following the introduction of conditional discharge by modern legislation recorded a much higher rate of positive moves. Location at baseline therefore no longer influenced positive or negative moves, the DUNDRUM-1 accounted for some of the variance but did not influence regression models, and the HCR-20 dynamic scale still accounted for most of the variance in positive moves though not for negative moves or conditional discharge. The DUNDRUM-3 (programme completion) remained significantly associated with positive moves and conditional discharge in binary logistic regression. Concordance or the lack of it for the DUNDRUM-4 recovery scales accounted for most variance in relation to negative moves.

### Limitations

The self-report ratings were obtained on a non-confidential basis. This may have caused some bias in the self-reports. However this would always be the case in collaborative recovery-oriented work. The degree of difference in the self-rated and clinician-rated scores despite this is therefore notable as a likely indicator of validity.

The apparent increase in concordance when those in low or minimal security are compared with those in high or medium security is a cross-sectional observation and may be explained either by selective placement over time, or by a process of change. A prospective study of this derived measure is required to clarify this. Another limitation of this study is that many of these scores are “dynamic” and therefore are likely to change over the period of follow up as patients recovered or relapsed. However this is also a strength. A further limitation is that while patients who agreed to complete the self rated measures were blind to the clinician rated scores, this was not possible for all items. For example in DUNDRUM-4 recovery item R4 “Leave” blinding was not possible as patients were aware of the level of leave they had at the time of rating.

A further limitation is that not all patients could participate in the self-rating exercise. For some this was because of lack of capacity, for others because of unwillingness to participate. This is a disadvantage of all self-report methods as compared to observer rating scales such as the HCR-20 and the clinician rated DUNDRUM-1, DUNDRUM-3 and DUNDRUM-4. This study therefore has less statistical power than earlier studies using only observer rating scales and some forms of statistical analysis were not possible.

## Conclusions

Self-rated scores for programme completion (DUNDRUM-3) and forensic recovery (DUNDRUM-4) did not predict moves between levels of therapeutic security or conditional discharge. Patient self-ratings do not have the predictive accuracy of clinician ratings. However as we had hypothesised, concordance between patient and clinician ratings on the DUNDRUM-3 programme completion and DUNDRUM-4 recovery scales improved as patients progressed along the recovery pathway of the hospital. Those who progressed to conditional discharge were those with the lowest (best) scores on the DUNDRUM-3 programme completion and DUNDRUM-4 recovery scales, and also the least differences between clinician ratings and self-ratings. It appears that concordance measured in this way is a useful index of recovery in a forensic setting. Lack of concordance (greater differences between clinician and patient ratings) appeared to be an independent predictor of negative moves. The means of improving this concordance is therefore of great interest and may in itself be an appropriate outcome measure for various forms of psycho-education and treatment programmes.

This study is part of a cycle of validation studies concerning measures of need for therapeutic security [26-28] and the related measurements of treatment completion and recovery in forensic settings [29-32]. Recovery is often regarded as a qualitative issue, a policy or process or ethos rather than a measure validated against ‘hard’ quantitative outcomes. The difficulties in implementing a recovery approach in forensic mental health have been reviewed recently [41] and the importance of user involvement has been emphasised [42]. We have demonstrated an approach to quantitative assessment of these processes as outcome measures and as structured professional judgement tools when making decisions about moves to less secure places and discharge. We have used a self-report version of the programme completion and recovery items and scales and derived a measure of concordance between clinician and patient ratings as a useful measure of progress towards discharge from forensic secure settings. The DUNDRUM toolkit was recently found in a review of routine outcome measures in forensic mental health services to fulfil three of four desirable criteria for such measures: functioning, risk and placement pathways [43]. We believe that the addition of these self-report scales and the calculation of concordance between clinicians and patients fulfils the fourth such criterion, recovery. The findings are of importance when clinicians and decision makers (courts, statutory mental health tribunals) consider evidence and make judgements on these matters. For future practice and research, approaches to service delivery should be developed with the goal of improving function. These should go beyond the content of treatment programmes and forensic recovery factors to include studies of the form, ethos and process of service delivery emphasising the growth of concordance between patient and clinician. Whether this would lead to shorter lengths of stay in secure forensic hospitals would be the necessary ‘hard’ outcome measure.

## Additional file

Additional file 1:
**The DUNDRUM Toolkit V1.0.27.** This latest version includes the self-rating versions of the DUNDRUM-3 programme completion and DUNDRUM-4 recovery scales.
