# Supplementary material for: Recovery and concordance in a secure forensic psychiatry hospital – the self rated DUNDRUM-3 programme completion and DUNDRUM-4 recovery scales
Source: BMC Psychiatry. 2015 Mar 28;15:61. doi: 10.1186/s12888-015-0433-x (PMC4397875; doi:10.1186/s12888-015-0433-x)
Supplement: Additional file 1: — The DUNDRUM Toolkit V1.0.27. This latest version includes the self-rating versions of the DUNDRUM-3 programme completion and DUNDRUM-4 recovery scales. [file 12888_2015_433_MOESM1_ESM.pdf]

DANGEROUSNESS UNDERSTANDING, RECOVERY and URGENCY MANUAL (THE DUNDRUM QUARTET)
V1.0.26 (01/08/13)
Four Structured Professional Judgement Instruments for Admission Triage, Urgency, Treatment Completion and Recovery Assessments

Harry G Kennedy, Conor O'Neill, Grainne Flynn, Pauline Gill, Mary Davoren

National Forensic Mental Health Service, Central Mental Hospital, Dundrum, Dublin 14, Ireland
And
Academic Department of Psychiatry, University of Dublin, Trinity College

Correspondence: kennedh@tcd.ie

© not to be copied or reproduced without permission.

Acknowledgements

This manual was written as a distillation of our training, our experience and our practice as forensic psychiatrists. Between us we have worked in four different countries so we hope that the structured professional judgement instruments contained here will work in a variety of health services and jurisdictions. With this in mind, the definitions, items and scales emphasise patient focused rather than institutional or local legal factors, in so far as possible. It is fashionable to say that a culture speaks through the authors of a text rather than the authors creating anything new. In this sense, any expertise we have drawn on is derived from a shared scientific culture, as described by Collins & Evans (2007). If this is the case then we hope that we are articulating a multi-disciplinary forensic mental health culture because many colleagues have contributed to this text through comments, criticisms and feedback, while many more have educated and enculturated us over the years, including many of our patients who are contributors to that culture. .

Those we should acknowledge as having directly helped in the drafting of this manual are numerous. We feel we must however acknowledge by name our colleagues at the Central Mental Hospital, Dundrum who have been involved in the development of this manual, including Paul Braham, Dearbhla Duffy, John Ferguson, Pauline Gill, Sally Linehan, Damian Mohan, Paul O'Connell, Helen O'Neill, Orla O'Neill, David Timmons and Brenda Wright.

This manual is particularly dedicated to our colleague Dr Charles Smith, formerly clinical director of the Central Mental Hospital at Dundrum for his wit, good humour, clinical skill and his ease and fluency as a communicator.

© not to be copied or reproduced without permission.

CONTENTS

Title page & authors	page i
Acknowledgments	page ii
Contents	page iii
**Overview**	page 1
Structured professional judgement	page 1
Complimentary relationship to risk	page 2
Evolution	page 2
Use of the DUNDRUM toolkit	page 3
Using this manual	page 4
**DUNDRUM-1: triage security items**	page 5
Facet system	page 7
Validity	page 8
Benchmarking validity	page 9
Items	page 10
**DUNDRUM-2: triage urgency items**	page 25
Validation	page 26
Items	page 28
**DUNDRUM-3: programme completion**	page 42
Five pillars of treatment	page 42
Item ratings and theory: Maslow, cycle of change, engagement	page 43
Validity	page 45
Items	page 46
**DUNDRUM-4: recovery items**	page 60
Items	page 61
**DUNDRUM toolkit Self Rated Version**	page 71
How to use this toolkit	page 72
Items	page 73
**References**	Page 87
**APPENDIX A: preadmission**	page 92
DUNDRUM-1 and DUNDRUM-2 forms	page 93
Preadmission weekly triage up-date forms	page 94
DUNDRUM-3 and DUNDRUM-4 six monthly up-dates	page 96

© not to be copied or reproduced without permission.

© not to be copied or reproduced without permission.

Overview

"Dangerousness is a dangerous concept" according to Shaw (1973). Scott (1977) quoted Shaw but went on to define dangerousness as the product of probability (risk) and gravity (seriousness). The admission criteria for special (high security) hospitals in Britain were defined as 'grave and immediate risk'. We believe that it is 'graveness', not just risk, that guides the decision to allocate a patient to high, or medium or low levels of therapeutic security. In recent decades the seriousness of the harmful behaviour under consideration has been largely unexamined in the research literature while a fruitful and scientifically productive literature has grown up around the assessment and management of risk of harm. For practical purposes, risk has often fallen substantially by the time a person is admitted from a waiting list to a therapeutically secure hospital, and it is the seriousness or gravity of the behaviour that appears to be the main determinant of the decision to allocate to a particular level of therapeutic security.

Our practice at the Central Mental Hospital, Dundrum, as at many similar units in other jurisdictions, is to hold a weekly meeting to consider referrals, transfers and discharges. The meeting is attended by all heads of discipline - medical/clinical director, director of nursing, heads of psychology, social work and occupational therapy, all consultant psychiatrists and all ward/unit managers (nurse managers). The meeting is usually chaired by the consultant psychiatrist who is 'on call' for the week. Although this is a large group, the meeting is a pivotal part of the management of any forensic mental health service. A key outcome of the weekly meeting is a triage decision concerning those accepted onto the waiting list and the prioritisation of those on the waiting list. Decisions are also taken about in-patients at this meeting such as imminent discharges and movements from areas of high therapeutic security, including admission units, to medium and on to minimum secure and pre-discharge areas, having previously been discussed as part of individual care and treatment planning in the multi-disciplinary teams.

The first four elements of this manual are structured professional judgment instruments to support the decision making process with a fifth self-report assessment for programme completion and recovery.

Structured Professional Judgment

Structured professional judgment is increasingly recognised as an effective way to improve the quality, consistency and transparency of decision making. Unstructured professional judgment is vulnerable to the criticism that it is arbitrary, and formal tests often show that it has poor inter-rater reliability. Actuarial check lists can claim greater scientific precision but may be excessively rigid, excluding obvious factors relevant to an individual case and generating scores that are reliable only for the specific populations in which they have been validated. Structured professional judgement instruments draw together factors for which there is research evidence of relevance. They also draw on the shared knowledge and language that make up the professional 'culture' of expertise, in the way that expertise is defined by Collins & Evans (2007). Structured professional judgement instruments provide a written set of definitions to facilitate training and inter-

© not to be copied or reproduced without permission.

rater reliability. They can be validated against criterion measures to show that the instrument does whatever function it claims to do. Structured professional judgement instruments merely serve to enhance the quality of the clinician's judgement as measured by consistency and reliability, to ensure that scientifically valid items are not forgotten, to make the decision making process transparent and to reduce the chance of serious error. The inspiration for the form of this manual is heavily indebted to the HCR-20 and to its family of related instruments.

Complimentary Relationship to Risk

We believe that the instruments defined in this manual are qualitatively different from the excellent and essential structured professional judgement instruments for the assessment of risk of violence. The DUNDRUM-1 triage security items are mainly static in nature and measure something that co-varies only to a small extent with the historical items of the HCR-20 (Webster et al 1997) and the background items of the S-RAMM (Bouch & Marshall 2003). The DUNDRUM-2 triage urgency items should be dynamic in nature, variable from one time to another but should co-vary only to a limited extent with the dynamic, clinical and risk management items of the HCR-20 or the current and future items of the S-RAMM. The DUNDRUM-3 programme completion items and DUNDRUM-4 recovery items co-vary to some extent with the protective items in the START and SAPROF (Abidin et al 2013).

We believe that the DUNDRUM toolkit of SPJ instruments should be used with the HCR-20, as they measure complimentary domains.

Evolution

Eastman & Bellamy's (1998) Admission Criteria for Secure Services Schedule (ACSeSS) is a set of criteria used in needs assessment which could be read as a structured professional judgement instrument. This identified seven domains relevant to need for placement in secure settings including the gravity of recent or past violent behaviour, the immediacy of any risk of violent behaviour in the community or in hospital, psychopathology that 'predicts' the above, specialised psychopathology that specifically determines anti-social behaviour – specialist forensic need; the likely duration of the admission, unpredictability and lastly how the case would be perceived by a criminal justice agency – a 'trump' factor that might determine admission to a higher level of security than other factors might indicate.

Kennedy (2002) compiled definitions for various levels of therapeutic security based on institutional characteristics but also provided clinical criteria for the allocation or stratification of patients to these various levels of therapeutic security. The same paper gave suggested criteria for the movement of patients down through the levels of therapeutic security, or along a pathway towards recovery.

Other approaches have included an algorithm based on severity of offence and legal category (Coid & Kahtan 2000); structured professional judgment instruments based on patient centered factors such as security needs, dependency needs, treatment needs, 'political' considerations and likely length of hospital stay using visual analogue scales

© not to be copied or reproduced without permission.

(Shaw et al 2001); a mixture of severity items and physical, staffing and procedural items (Sugarman & Walker 2004); and security centered institutional factors such as physical security, relational security and procedural security with detailed item definitions (Collins & Davies 2005). The last two of these have in common a rating system designed to match levels of security, from 0 to 4. An actuarial tool based on risk factors which contained only one item reflecting seriousness of violence had a moderate receiver operating characteristic but modest predictive power (Brown & Lloyd, 2002 & 2008).

Use of the DUNDRUM Toolkit

We have collated the material referred to in the previous paragraph along with our own experience and research to draft the four sets of items in this handbook. The first four elements of this manual are not intended to be used as actuarial scores that provide ‘cut-off’ points above or below which a particular decision on allocation or stratification is determined. But structured professional judgment instruments are not meant to generate scores or thresholds that replace the discretion of the clinical decision maker. Although validated like actuarial scores, the advantage of a structured professional judgement instrument is that it ensures transparency and consistency of decision making. A high score on one item might be enough to decide the level of therapeutic security needed. Conversely, a moderate or high total score made up of numerous ‘2’s and occasional ‘3’s might best be managed in low security or even in the community. These items are intended to guide clinical decision making but not to bind the decision maker.

<u>Mental Disorder as an Essential Pre-Requisite</u>

An essential caveat underpinning all that follows is that this manual consisting of four instruments or collections of items, is intended for use only when decisions are made about those who have a mental disorder, as established by clinical assessment and diagnosis. While mental disorder need not be an essential qualifying condition for many forms of therapy, it is an essential pre-requisite for admission to the therapeutically secure hospital and community mental health services for which this toolkit is designed. Remission (absence of symptoms) is not however the same as recovery and remission is not in itself sufficient for absolute discharge. Therapies for those who do not have a mental disorder and have intact mental capacities should for ethical reasons be provided voluntarily and without inducement or duress. In practice, for offender populations this should mean providing such therapies within the prison or community / probation setting rather than in a secure hospital or community forensic mental health service.

<u>Pre-Admission assessment</u>

It is intended that the DUNDRUM-1 Triage Security and DUNDRUM-2 Triage Urgency items in this manual might be used as part of the pre-admission assessment of those presenting to prison in-reach and court liaison / diversion services, as part of pre-sentence assessments when admission to a mental health service or community mental health team is under consideration, and when assessing anyone referred for admission or transfer to a therapeutically secure service. As outlined above, we recommend that these instruments should be used with the HCR-20 or other structured professional judgement tools for the assessment of risk. These instruments are not intended for the assessment of risk.

© not to be copied or reproduced without permission.

Moves Along the Recovery Pathway

The DUNDRUM-3 Programme Completion and DUNDRUM-4 Recovery Items should be of assistance when making decisions about evidence of change and readiness for a move to less secure or community settings.

As a general principle, we believe this manual could be adapted to perform a similar function in any mental health service including general and specialist groups and settings catering for life cycle stages or developmental needs, in much the same way that the Camberwell Assessment of Need (CAN) or Health of the Nation Outcome Scales (HONOS) have been adapted for different patient groups.

Further Applications

The various elements of the manual generate a useful data set for audit projects concerning accessibility and equitability of services, quality and outcomes.

Admission and Discharge Thresholds

The threshold for admission to a given level of therapeutic security may change over time. In the U.K. in the 1980s, almost all who were severely mentally ill and who killed were admitted to one of the ‘Special’ (High Security) Hospitals. By the end of the 1990s, most such persons were admitted to medium secure units. This change was brought about in part by an intended reform of practice and in part as an unintended consequence of the closure of a large proportion of the Special Hospital beds, so that admission thresholds had to rise. In general the availability of secure beds at any level, combined with the availability of alternatives at higher and lower levels of therapeutic security, will determine the threshold for admission to that level. This availability is largely determined by the dynamic effects of changes in average length of stay and the numbers discharged each year, while the actual number of beds at a given level of security has a static role. For this reason, the ‘Recovery’ items should be rated at the earliest opportunity, ideally at the same time as the first rating of the Triage items and these should be regarded as inseparable.

Using This Manual

We strongly recommend that ratings should only be completed with the full manual open – the definitions are essential if any consistency or reliability is to be achieved. The ratings are likely to be most accurate if completed collaboratively by a multi-disciplinary team. The patient / service user should also be involved in the process as a part of the therapeutic transaction if possible. In this revision of the handbook, the self-report versions of the DUNDRUM-3 and DUNDRUM-4 are set out. The decision regarding actual admission, transfer or discharge remains the responsibility of the appropriate clinician and legal decision makers where relevant.

It is too early as yet to describe systematic training, but we recommend the use of vignettes.

© not to be copied or reproduced without permission.

DUNDRUM-1: TRIAGE SECURITY ITEMS

The triage items should be distinguished qualitatively from the items included in structured professional judgement tools for risk assessment such as the HCR-20. The Triage items are divided here into DUNDRUM-1 Triage Security items and DUNDRUM-2 Triage Urgency items.

The triage items are all predicated on there being an established mental disorder present, whether mental illness, mental impairment or dementia, or any other legal category in the jurisdiction in which the instrument is to be used e.g. psychopathic disorder in England & Wales. In accordance with international conventions such as COE Rec(10)2004, intoxication and social deviance are excluded from mental disorder. It is made clear in the definitions that absence of a mental disorder leads to a 'zero' rating. Diagnosis of mental disorder can in almost all cases be established by a pre-admission assessment. This should always be carried out by the admitting service, though it is good practice to obtain an independent medical certification before completing a compulsory admission order and in many jurisdictions this is a legal requirement. In the absence of a mental disorder, there may still be a need for an assessment of security need, but this may be better carried out by professionals other than the mental health team e.g. using the LSI-R (Andrews & Bonta 1995).

The purpose of the triage security items is to structure the decision making process when deciding what the appropriate level of therapeutic security might be for a person who is in need of admission to hospital from the criminal justice system – court or prison, or who has been referred for transfer to a more secure hospital or unit from a community mental health service. The DUNDRUM-1 triage items therefore are not intended to be used as a guide to the risk of future violence – the HCR-20 and other structured professional judgment and actuarial tools have already been validated for that purpose. Nor are the DUNDRUM-1 triage items intended to produce an actuarial score relating to fixed admission thresholds. These items should be regarded as a means of structuring the decision making process in accordance with factors that are relevant, in a way that is transparent and will lead to greater consistency. They may facilitate benchmarking between services and jurisdictions.

In general, a person who is mostly rated '4' on these Triage Security Items is likely to require conditions of high therapeutic security at least for the early part of an admission to hospital; a person who is mostly rated '3' is likely to need conditions of medium security, at least initially; a person who is mostly rated '2' will benefit from treatment in conditions of psychiatric intensive care (acute low security), whether for a short or longer period; a person mostly rated '1' should be safely treated and cared for in an open in-patient setting; a person mostly rated '0' may be cared for in a community setting, including home treatment, crisis houses, high support community residences and other options. A person rated '0' could also be followed by a prison in-reach mental health team. This does not preclude admission to hospital including secure placements, and / or the use of mental health legislation where appropriate. Further definitions of the various levels of therapeutic security have been defined elsewhere (Kennedy 2002)

© not to be copied or reproduced without permission.

Under the legal structures of some jurisdictions, courts have the power to determine that a person shall be admitted to a forensic mental health unit. This is often grounded in legislation creating a special status for selected secure hospitals, variously described as Special Hospitals (England & Wales), the State Hospital (Scotland), a designated centre (Ireland, Ontario) and other legal variants. The DUNDRUM-1 is designed as a structured professional judgement tool to assist the clinicians who act as expert witnesses or who are required to fulfil statutory obligations in advising the courts regarding the appropriateness of committal to a secure psychiatric facility. The Triage Security items may also be used as an audit tool for the appropriateness of such placement recommendations and orders.

The DUNDRUM-2 Triage Urgency items are intended to provide a structure for deciding who on a waiting list for admission to a given level of security is the most urgent. In general, a higher score indicates the more urgent need. However at the time of drafting this first version, it is not clear that the items are logically or ethically simply additive. As clinicians, the authors are strongly of the opinion that clinical urgency should always take precedence over other non-clinical factors. In practice, there may be times when a legal obligation over-rules a clinical priority. This may have adverse health consequences for the more clinically urgent case. It is the responsibility of the clinicians and clinical managers to ensure that the legal decision maker is fully aware of the consequences of such exercise of legal power.

As for all structured professional judgement tools, the decision makers are not bound by the ‘result’ of the assessments. One highly rated item may be enough to require admission to the highest levels of therapeutic security given an individual context. Other factors that are not included in this toolkit may become relevant in an individual case.

While not directly relevant to the work of a therapeutic institution or service, the following prison / corrections perspective on the need for different levels of security derived from the Learmont (1995) report, is important to bear in mind, since some determined criminals may seek to use transfer to hospital as a means of easing their escape –

© not to be copied or reproduced without permission.

Facet System for Classification Criteria [1]

	**Danger to public**	**Escape risk**	**External resources**
**1**	Not dangerous	Trusted	No resources
**2**	Dangerous	Opportunistic	Outside resources
**3**	Highly dangerous	Determined and skilled	Outside resources and valued member of a terrorist or organised crime group

Classification Guidelines:

	Danger to public	Escape risk	External resources	Total
Category A Exceptional Risk	3	3	3	9
Category A High Risk	332, 323, 233			8
Category A Standard Risk	322, 331, 313, 232, 133, 223			7
	321, 312, 231, 222, 132, 213, 123			6
Category B	311, 221, 212, 131, 122, 113			5
Category C	211, 121, 112			4
Category D	111			3

[1] Learmont Report Appendix N, ppN2-5.
Canter D (ed) (1985) Facet Theory: Approaches to Social Research. New York:Springer Verlag.
Shye s, Elizur D, Hoffman M (1994) Content Design and Intrinsic Data Analysis in Bhavioural Research. California: Sage.

© not to be copied or reproduced without permission.

VALIDITY

The scores for the eleven item DUNDRUM-1 triage security instrument have very good internal consistency and differentiated patients referred from a remand prison according to the level of security to which they were eventually admitted (Flynn et al 2011a).

Those not followed up (n=159) could be distinguished from others (n=87) by the receiver operating characteristic (ROC area under the curve (AUC)=0.893. SEM=0.026, p<0.001. Those diverted from prison/court to hospital (n=30 including local open units, PICU or MSU) could be distinguished from those not diverted from prison (n=216) AUC=0.984, SEM = 0.007, p<0.001. A cut-off score of 5.5 yielded a sensitivity of 97% and a specificity of 91%. However, the sensitivity and specificity of higher scores as indicators of the need for open ward conditions, PICU or medium secure conditions requires more data.

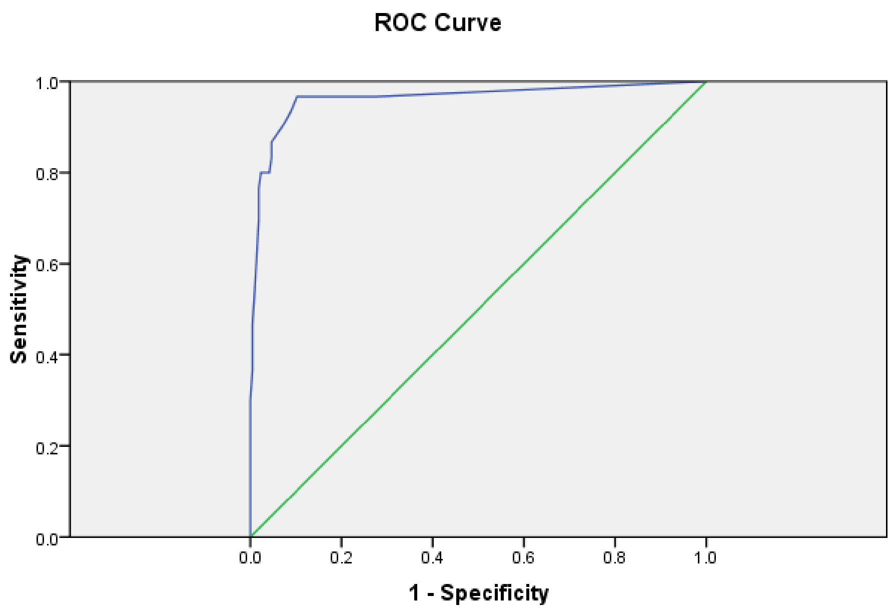

Diagonal segments are produced by ties.

The Receiver operating characteristic for those admitted to a hospital (n=30) via a prison in-reach and court liaison service, compared to those not diverter from prison (n=216).

Area Under the Curve

Test Result Variable(s):TOTALSCORE

Area	Std. Error[a]	Asymptotic Sig.[b]	Asymptotic 95% Confidence Interval	
			Lower Bound	Upper Bound
.984	.007	.000	.971	.997

© not to be copied or reproduced without permission.

The same study (Flynn et al 2011a) showed that the DUNDRUM-1 score distinguished between the levels of therapeutic security to which remand prisoners were eventually admitted. Each of the 11 items also corresponded with eventual placement, with the two suicide and self harm items performing marginally less well than other items.

In subsequent studies the DUNDRUM-1 has been used to allow benchmarking of in-patient need for therapeutic security (Davoren et al 2013a) and risk (Abidin et al 2013). The DUNDRUM-1 was shown to be a robust predictor of moves between levels of therapeutic security, along with the HCR-20 (Davoren et al 2013a) but it was not a predictor of conditional discharge to the community (Davoren et al 2013b). The study of moves between levels of therapeutic security appears to confirm that the DUNDRUM-1 measures something (‘seriousness’) that is complimentary to and independent of risk.

Benchmarking Validity

We now suggest that when benchmarking the need for therapeutic security for a group or cohort, rather than dividing the group mean DUNDRUM-1 11 item score by 11, it is better to divide the group’s mean score for nine items by nine, omitting items concerning suicide and self-harm TS2 and TS4.

© not to be copied or reproduced without permission.

Triage Security Item 1: Seriousness of Recent Violence

The seriousness or gravity of a risk is an aspect of dangerousness that is often missed by risk assessment tools. This item should be distinguished from the later item dealing with public confidence issues. Scott (1977), in an influential early paper on risk assessment, defined 'dangerousness' as a product of probability (risk) and the gravity of the risk in question. A person may be at high probability of some minor act, or at a low probability of some very serious act, such as homicide. The term 'concern' may be more appropriate for this factor than 'dangerousness' and has been employed in some recent scholarship and research (e.g. James et al 2010). Assessing the gravity of violence risk is therefore a legitimate element in the rational triage of those requiring psychiatric treatment. Eastman & Bellamy (1998) identified the seriousness of violent acts as the first element of a structured professional judgement manual for auditing security needs. Coid & Kahtan (2000) using a classification of seriousness of the most recent offence showed that this was one of the elements of an algorithm correctly describing the allocation of patients to various levels of therapeutic security.

The scientific evidence for specialisation in some offending careers is easily overshadowed by evidence that most offenders are diverse in their offending behaviour. Evidence of specialisation is strongest for sexual offences (Stander et al 1989, Grubin et al 2001). Tracy et al (1990) found that the average seriousness of offences increased with recidivism. Specialization also increased as offenders became older and with each successive offence. Offenders released from prison in the USA were 53 times more likely than the general population to be rearrested for homicide over the next three years, while those released from prison whose most recent offence was homicide were 1.4 times more likely than other offenders to be rearrested for homicide, and many times more likely than the general population. Similar specialization emerged for all violent offences, rape, other sexual assaults, robbery, property offences and fraud (Langan & Levin 2002). Similar ‘specialization’ can be shown for mentally disordered arsonists (Rice & Harris 1996) and stalkers (Mullen et al 2009) amongst others. See also Walker & McCabe (1973, vol 2 p194). Specialization and escalation are real phenomena, comparable to suicide research regarding ‘preferred method’ (Appleby et al 2001) and ‘lethality’ (Beautrais 2001).

Where there is a recent history of life-threatening violence, higher levels of therapeutic security will be required. This is not however the only determinant of the level of therapeutic security required, and other factors, as listed in this guide, should always be considered also. The seriousness of the risk of suicide is recognised as an important determinant of risk of suicide (see for example the S-RAMM), but in the context of this instrument we take seriousness as a guide to the level of therapeutic security required.

It follows that these two items are rated as ‘historical’. They should rely on behaviour for which there is at least prima facie evidence – charges pending, charges brought, facts proven on the balance of probabilities (civil standard), facts proven beyond reasonable doubt (criminal standard, e.g. facts proven but unfit to stand trial) convictions in court (beyond reasonable doubt). Assaults in hospital for which no charges were brought

© not to be copied or reproduced without permission.

should be documented according to the date and time of contemporaneous description in the hospital notes.

NB All previous violence must be rated, even if the person was not mentally disordered at the time of past violence. Rate on the most serious violent act known.
NB If there is no current mental disorder (broadly defined), the correct rating is zero (0), because the person is not in need of psychiatric admission or follow up.

Coding: TS1. Seriousness of Violence

4	4.1 Homicide **or**<br>4.2 Stabbing penetrates body cavity **or**<br>4.3 Fractures skull **or**<br>4.4 Strangulation **or**<br>4.5 Serial serious (e.g. penetrative, indictable) sexual assaults **or**<br>4.6 Kidnap **or** torture **or** poisoning.
3	3.1 Use of weapons to injure **or**<br>3.2 Arson endangering life **or**<br>3.3 Assaults causing concussion **or**<br>3.4 Fractures to long bones **or**<br>3.5 Stalking with threats to kill **or**<br>3.6 Single serious sexual assault, (indictable).
2	2.1.1 Repetitive assaults causing injury such as bruising **and**<br>2.1.2 That cannot be prevented by two-to-one nursing in open conditions **or**<br>2.2 Less serious sexual assaults, (summary offence)
1	1.1 Minimal degrees of violence **and**<br>1.2 Minimal threat to life.
0	0.1 No previous violence, **or**<br>0.2 No current mental disorder (mental disorder includes adjustment reaction)

Note: for the purposes of item TS3, a rating of ‘3’ or ‘4’ is ‘serious violence’ and a rating of ‘1’ or ‘2’ is ‘less serious violence’.

Information Quality: 0=no information; 1=staff observation only; 2=interview and staff observation; 3=family informants; 4=medical or police records.

© not to be copied or reproduced without permission.

Triage Security Item 2: Seriousness of Self-Harm

NB the previous item TS1 needs little adaptation to be applied to attempted suicide and self-harm. The aim here is to emphasise the seriousness of the attempt, with added weight given to the current suicidal intent. Although these factors can be found in risk assessment instruments for suicide, we are concerned here to assess the seriousness or gravity of the harm. For a fuller account of the risk of suicide and self harm see the S-RAMM, a structured professional judgement instrument (Bouch & Marshall 2003, Ijaz et al 2009, Fagan et al 2009)..

NB If there is no current mental disorder (broadly defined), the correct rating is zero (0), because the person is not in need of psychiatric admission or follow up.
NB All previous self-harm must be rated, even if the person was not mentally disordered at the time of past self-harm. Rate on the most serious self-harming act known.
NB If there is no current mental disorder (broadly defined), the correct rating is zero (0), because the person is not in need of psychiatric admission or follow up.

Coding: TS2: Seriousness of Self-Harm

4	4.1 Near miss attempts at suicide – hanging with loss of consciousness, overdoses requiring ventilation or organ support, jumping from significant heights **or**<br>4.2 Arson (e.g. fire in own cell/bedroom) requiring prolonged hospital treatment
3	3.1 Use of potentially lethal means such as ligatures, arson, jumping to injure self
2	2.1 Repetitive self-harm causing non-life-threatening injury **and**<br>2.2 Cannot be prevented by two-to-one nursing in open conditions
1	1.1 Self harm of minimal severity **and**<br>1.2 Minimal actual threat to life
0	0.1 No previous self-harm, **or**<br>0.2 No current mental disorder (mental disorder includes adjustment reaction)

Note: for the purposes of item TS4, a rating of ‘3’ or ‘4’ is ‘serious self-harm’ and a rating of ‘1’ or ‘2’ is ‘less serious self-harm’.

Information Quality: 0=no information; 1=staff observation only; 2=interview and staff observation; 3=family informants; 4=medical or police records.

© not to be copied or reproduced without permission.

Triage Security Item 3: Immediacy of Risk of Violence due to Mental Disorder

The immediacy of a risk determines the extent to which high, medium or low levels of supervision are currently required. In higher levels of therapeutic security, higher staff-to-patient ratios ensure closer monitoring and greater opportunities for early de-escalation of any threat of violence. “Serious violence” here refers to violence rated ‘3’ or ‘4’ on item TS1 ‘seriousness of violence’.

There are various ways in which a risk may be immediate – an unassessed risk due to a mental disorder is for practical purposes unpredictable, and should therefore be regarded as immediate. Those with pervasive anger and resentment often have heightened sensitivity and may be explosive or provoked in response to minimal or mistakenly perceived ‘provocations’. Paranoid psychoses, acute schizophrenia or manic states may all be associated with such angry, sensitive mental states. A person who has a mental disorder co-morbid with intoxication or unmanaged withdrawal is likely to be labile in mood and similarly impulsive and unpredictable.

Scales such as the DASA can be used to reliably rate the warning signs for immediate or short term risk of violence.

An acute relapse of a mental illness leading to such problems may be time limited. Such episodes may resolve with treatment in three to six months and may be managed in lower secure settings designed for short term care. Others may be anticipated to remain at risk for longer periods and may therefore require treatment in settings intended to cope with longer term continuing risk.

Coding: TS3. Immediacy of Risk of Violence due to Mental Disorder

NB If there is no current mental disorder (broadly defined), the correct rating is zero (0), because the person is not in need of psychiatric admission or follow up.

4	4.1 Still in the mental state that led to serious violence.
3	3.1 Partially recovered from mental state that led to serious violence
2	2.2 Still in mental state that led to less serious violence
1	1.1 Partially recovered from mental state that led to less serious violence **or**<br>1.2 Non-violent offence
0	0.1 No abnormality of mental state **and /or**<br>0.2 No violence. (mental state includes current adjustment reactions)

Information Quality: 0=no information; 1=staff observation only; 2=interview and staff observation; 3=family informants; 4=medical or police records.

© not to be copied or reproduced without permission.

Triage Security Item 4: Immediacy of Risk of Suicide

Like the previous item TS3, this is a dimension which may influence the initial triage decision but should not be regarded as enduring – the rating can be revised down or up. See also the S-RAMM (Bouch & Marshall 2003, Ijaz et al 2009, Fagan et al 2009). Here ‘serious self harm’ means an attempt rated ‘3’ or ‘4’ on item TS2 and ‘less serious self harm’ means an item rated ‘1’ or ‘2’.

As for immediacy of risk of violence, an acute relapse of a mental illness leading to such problems may be time limited. Such episodes may resolve with treatment in three to six months and may be managed in lower secure settings designed for short term care. Others may be anticipated to remain at risk for longer periods and may therefore require treatment in settings intended to cope with longer term continuing risk.

Coding: TS4. Immediacy of Risk of Suicide

NB If there is no current mental disorder (broadly defined), the correct rating is zero (0), because the person is not in need of psychiatric admission or follow up.

4	4.1 Still in the mental state that led to serious self harm (high lethality).
3	3.1 Partially recovered from mental state that led to serious self harm (high lethality)
2	2.1 Still in mental state that led to less serious self harm
1	1.1 Partially recovered from mental state that led to less serious self harm
0	0.1 No current abnormality of mental state (mental state includes symptoms of adjustment reaction) **and /or**<br>0.2 No history of suicidal or self harming behaviour.

Information Quality: 0=no information; 1=staff observation only; 2=interview and staff observation; 3=family informants; 4=medical or police records.

© not to be copied or reproduced without permission.

Triage Security Item 5: Specialist Forensic Need

There are persons for whom the recorded seriousness of violence and imminence of risk are not enough to fully describe the need for specialist forensic care and treatment.

When a person has a previous history of treatment in conditions of high or medium security, it may be presumed that on relapse they will need to return to the highest levels of security they have previously been allocated to. This has limited if any validity, and should be subjected to a structured reassessment of the current need as described by the totality of this guide. Where there is any doubt, it is better to err on the side of caution if readmitting, and in the first instance readmit to a lower level of therapeutic security than before. One of the practical indicators of the level of therapeutic security currently needed is that the person has demonstrably exceeded the safe capacity of a well-organised therapeutically secure service at a lower level. Evidence of this might include serious adverse incidents in the current placement (at a lower level of therapeutic security) or loss of confidence amongst the staff at the lower level.

There are problems for which treatment can only continue in a therapeutically safe and secure environment. These are usually problems for which the therapist might be at risk in the course of treatment. Patients who incorporate clinicians into their delusional systems, patients in whom sadistic or expressively violent patterns of behaviour are prominent, arsonists or others may require a high level of therapeutic security for treatment to proceed. For practical purposes, specialist treatment programmes for such problems can often only be delivered in conditions of therapeutic security, at least initially.

© not to be copied or reproduced without permission.

Coding: TS5. Specialist Forensic Need

NB If there is no current mental disorder (broadly defined), the correct rating is zero (0), because the person is not in need of psychiatric admission or follow up.

4	4.1 Sadistic, paraphilias associated with violence **or**<br>4.2 Exceeds capacity of medium security.
3	3.1 Arson, jealousy, resentful stalking **or**<br>3.2 Exceeds capacity of PICU / low secure unit.
2	2.1 Current mental state associated with violence **and**<br>2.2 May include crisis or recall of former medium / high security patient
1	1.1 Cannot cooperate with voluntary treatment, **and**<br>1.2 Compliant when detained.
0	0.1 No history of mental disorder (mental disorder includes current adjustment reaction), **or**<br>0.2 Co-operates with voluntary treatment **and**<br>0.3 Integrates into community mental health services **and**<br>0.4 Consents to all interventions recommended.

Information Quality: 0=no information; 1=staff observation only; 2=interview and staff observation; 3=family informants; 4=medical or police records.

© not to be copied or reproduced without permission.

Triage Security Item 6: Absconding/Eloping

One of the uses of therapeutic security is to prevent absconding (referred to in North American literature as ‘eloping’). Clinical risk management indications for preventing absconding include preventing suicide or self harm, and preventing harm to others. Learmont (1995) provides an algorithm for identifying those in need of increasing levels of security to prevent escape from within a secure setting. One of the factors identified by Learmont is ‘trust’.

This item should be rated conservatively – those who can safely be cared for at home or in an open setting with close nursing observations e.g. to prevent self harm or suicide, should not be moved to more secure settings.

Legal obligations may be imposed over clinical considerations at times, e.g. to ensure that those facing long sentences or currently serving long sentences do not abscond.

© not to be copied or reproduced without permission.

Coding: TS6. Absconding/Eloping

NB If there is no current mental disorder (broadly defined), the correct rating is zero (0), because the person is not in need of psychiatric admission or follow up.

4	4.1 Currently has not demonstrated capacity for trust in relation to absconding **and**<br>4.2 Past history of absconding from custody at medium or high security levels **or**<br>4.3. Is capable of planning, deception, corruption or coercion in order to abscond/escape **or**<br>4.4 May be helped to abscond/escape by third parties.
3	3.1.1 Currently pre-sentence **and**<br>3.1.2 Currently facing a serious charge **or**<br>3.2.1 Currently serving a long sentence, **and**<br>3.2.2 Capable of planning and deception in order to abscond/escape.
2	2.1 Current risk of impulsive (opportunistic) absconding/escaping only **and**<br>2.2 absconding could be prevented by admission to PICU.
1	1.1 If absconded or broke off contact, would not present an immediate danger to the public **and**<br>1.2 Would not present a grave danger (whether immediate or not) to the public **and**<br>1.3 Would not present a danger to specific potential victims.
0	0.1 No history of mental disorder (mental disorder includes current adjustment reaction) **or**<br>0.2 Will not break off contact with mental health team in the community or prison in-reach mental health service.

Information Quality: 0=no information; 1=staff observation only; 2=interview and staff observation; 3=family informants; 4=medical or police records.

© not to be copied or reproduced without permission.

Triage Security Item 7: Preventing Access

There may be reasons why it is necessary to protect the person concerned from specific stressors e.g. the ready availability of drugs or intoxicants if these might otherwise be readily available, to prevent access to weapons, or to protect specific individuals or categories of person. This may include the ability to monitor and under certain defined circumstances to block communications e.g. in relation to the victims of stalking or threats, to other vulnerable or potential victims and access to pornography, violent material or other threatening material.

Coding: TS7. Preventing Access

NB If there is no current mental disorder (broadly defined), the correct rating is zero (0), because the person is not in need of psychiatric admission or follow up.

4	4.1 Requires some restriction and monitoring of access to intoxicants, weapons, communications, media and access to vulnerable persons – will misuse if access is possible **and**<br>4.2 Has the capacity to obtain contraband, media, communications etc by means of planning, deception, corruption, coercion **or**<br>4.3 By means of the help of third parties, **or**<br>4.4 Needs protection from well-organised gangs/third parties
3	3.1 Requires some restriction and monitoring of access to intoxicants, weapons, communications, media and access to vulnerable persons – will misuse if access is possible **and**<br>3.2 Is capable of some planning or deception to gain access to contraband or forbidden media / communications. **or**<br>3.3 Needs to be separated from others he might have feuds / grudges against or who might have grudges against him
2	2.1 Requires some restriction and monitoring of access to intoxicants, weapons, communications, media and access to vulnerable persons **and**<br>2.2 Is sufficiently limited in PICU / acute low security due to impulsive, unplanned nature of actions.
1	1.1 Will comply with all aspects of risk management regarding restricted and monitored access to intoxicants, weapons, communications, media and access to vulnerable persons or potential victims while in hospital.
0	0.1 No history of mental disorder (mental disorder includes current adjustment reaction) **OR**<br>0.2 Can be trusted not to misuse intoxicants, weapons, communications, media or access to vulnerable persons without the need for imposed restrictions and monitoring in the community.

Information Quality: 0=no information; 1=staff observation only; 2=interview and staff observation; 3=family informants; 4=medical or police records.

© not to be copied or reproduced without permission.

Triage Security Item 8: Victim Sensitivity / Public Confidence Issues

An awareness of the risks to others is an important part of the triage decision. Risks to others include those who have been the victims of explicit threats to kill or persistent unwanted attention (stalking). High-risk relationships may be relevant here, even when the third party wishes to have or resume full contact (battered spouses, children or parents).

Stranger victims or neighbours may object to the return of the person to their vicinity because of their fears or subjective discomfort.

Social and community considerations may also be relevant - local notoriety, media interest and the risk of revenge or reprisals against the person may all be relevant.

Coding: TS8. Victim Sensitivity / Public Confidence Issues

NB If there is no current mental disorder (broadly defined), the correct rating is zero (0), because the person is not in need of psychiatric admission or follow up.

4	4.1 Has national / media notoriety, **or**<br>4.2 Has made explicit credible threats to kill named individuals
3	3.1 Significant local notoriety or local media interest. **or**<br>3.2 Predictable potential victims (including vulnerable family members or high risk relationships);
2	**Either**<br>2.1 Short-term **or** Enduring<br>2.2.1 Family sensitivities **or**<br>2.2.2 Victim sensitivities.
1	No long term local sensitivity or notoriety
0	0.1 No history of mental disorder (mental disorder includes current adjustment reaction) **or**<br>0.2 No local victim sensitivities or community sensitivities **and**<br>0.3 no high risk relationships

Information Quality: 0=no information; 1=staff observation only; 2=interview and staff observation; 3=family informants; 4=medical or police records.

© not to be copied or reproduced without permission.

Triage Security Item 9: Complex Needs Regarding Risk of Violence

This item can best be described as a qualitative ‘profile’ of the factors relevant to risk of violence, in so far as this relates to the level of therapeutic security required for safety and specialist treatment programmes to alleviate the combination of problems. As outlined in the introduction, this tool is intended to assist decision making regarding the level of security required..

The rating chosen here offers the opportunity to use the ‘Historical’ items of risk assessment instruments such as the HCR-20 as they were intended, as a guide to structured professional judgement. The ratings described below offer ‘profiles’ based on the most widely used static, background or historical risk factors to rate increasing complexity of treatment needs and need for therapeutic security. Major mental illness may be taken as defined in HCR-20 H6. Violence or harm may be taken as defined in HCR-20 H1.

NB This pattern needs little adaptation to describe risk of suicide (see for example S-RAMM). However a risk of suicide in the absence of a significant risk of violence is always manageable in open hospital or low-secure settings. Medium or higher levels of therapeutic security are required for prison to hospital transfers only when other factors intervene such as absconding risk (TS6) or institutional behaviour (TS10).

© not to be copied or reproduced without permission.

Coding: TS9. Complex Needs Regarding Risk of Violence

4	4.1 Current and / or previous serious violence **not** confined to the context of active symptoms of major mental illness; **or**<br>4.2 Co-morbid high score on the PCL-R or PCL-SV (threshold as in HCR-20 H7);
3	3.1 Previous serious violence in the context of major mental illness **and**<br>3.2 Substantial co-morbidity (complex problems) – i.e. major mental illness with one of the following - **either**<br>3.3 Severe substance misuse problems (e.g. daily misuse or weekly binges) **or**<br>3.4 Severe personality disorder (persistent even when mental illness and substance misuse are in remission) **or**<br>3.5 Other relevant significant historical/background risk factors (e.g. intellectual disability, acquired brain injury).
2	2.1 Previous violence/harm **and**<br>2.2 Current / recent violence in the context of major mental illness **and**<br>2.3 Co-morbid problems if present are minor / not prominent.
1	NB No history of violence.<br>1.1 Major mental illness is the only definite background/static risk item identified,<br>1.2 **may have** co-morbidity (substance misuse, personality disorder)
0	0.1 No history of major mental illness.<br>0.2 Other factors may be present, **but** this profile is best managed within the criminal justice system – see LSI-R or similar.

Information Quality: 0=no information; 1=staff observation only; 2=interview and staff observation; 3=family informants; 4=medical or police records.

© not to be copied or reproduced without permission.

Triage Security Item 10: Institutional Behaviour

Berecochea and Gibbs (1991) found that behaviour during previous periods in custody was one of the classification factors relevant to the appropriate level of security for individuals, at least in prison. The behaviours rated here may also be relevant to moves between levels of therapeutic security.

© not to be copied or reproduced without permission.

Coding: TS10. Institutional Behaviour

NB If there is no current mental disorder (broadly defined), the correct rating is zero (0), because the person is not in need of psychiatric admission or follow up.

4	4.1 Hostage taking in hospital or other secure institution **or**<br>4.2 Co-ordination of disturbances in hospital or other institution (i.e. a prime mover in such behaviour) **or**<br>4.3 Necessity to separate from other specific persons to prevent harm to others (e.g. feuds) **or**<br>4.4 Fashioning weapons or other contraband within the secure setting **or**<br>4.5 Sexually predatory/coercive behaviour towards vulnerable fellow-patients or in-mates.
3	3.1 Fire setting in hospital **or**<br>3.2 Barricading (without hostages) **or**<br>3.3 Roof-top protests in hospital or other secure settings as follower or without accomplices **or**<br>3.4 Sexually active with vulnerable fellow patients (non-coercive) **or**<br>3.5 High risk threats of serious violence to staff and/or in-mates and/or patients **or**<br>3.6 May have a history of previous serious violence while in hospital.
2	2.1 Impulsive fire setting or other high risk behaviour in the community which can be managed in hospital with observation and behavioural programme **or**<br>2.2 Bullying or coercive behaviour towards vulnerable fellow patients **or**<br>2.3 Threatening to staff e.g. while incorporating into delusions **or**<br>2.4 May have a pattern of previous less serious violence while in hospital.
1	1.1 Socially embarrassing, undignified, disruptive, challenging or threatening behaviour when in the community **or**<br>1.2 Behaviour that might lead to arrest for public order or minor / non-violent offences **or**<br>1.3 Behaviour that might cause damage to patient’s social network **but**<br>1.4 No habitual pattern of violence in hospital.
0	0.1 No history of mental disorder (mental disorder includes current adjustment reaction) **or**<br>0.2 None of the problem behaviours listed above for a proportionate period of time, with evidence of change.

Information Quality: 0=no information; 1=staff observation only; 2=interview and staff observation; 3=family informants; 4=medical or police records.

© not to be copied or reproduced without permission.

Triage Security Item 11: Legal Process

Note that the least restrictive option possible and acceptable to all should be preferred as the rating here. ‘All parties’ implies that the court should be satisfied with the proposed arrangement since the court is likely to have a veto.

Coding: TS11. Legal Process

NB If there is no current mental disorder (broadly defined), the correct rating is zero (0), because the person is not in need of psychiatric admission or follow up.

4	4.1 Only admission to a forensic secure centre is legally possible
3	3.1 Only admission to a forensic secure centre is acceptable to all parties.
2	2.1 Admission to low secure unit (e.g. PICU) legally possible **and**<br>2.2 Acceptable to all parties
1	1.1 Admission to local approved centre (e.g. open admission ward) legally possible **and**<br>1.2 Acceptable to all parties
0	0.1 No history of mental disorder (mental disorder includes current adjustment reaction) **or**<br>0.2 Community placement (out patient) legally possible and acceptable to all parties

Information Quality: 0=no information; 1=staff observation only; 2=interview and staff observation; 3=family informants; 4=medical or police records.

© not to be copied or reproduced without permission.

DUNDRUM-2: Triage Urgency Items

These items are intended to provide a structured professional judgement instrument for prioritising those admitted from the waiting list to a therapeutically secure service. Those placed on the waiting list should be determined by the DUNDRUM-1 security triage items, though even this is a matter for clinical judgement and flexibility in the light of the patient's best interests.

The allocation of places is not a simple matter of first-come, first-served, assigning to each a place on the waiting list determined by the date the individual is first accepted onto the waiting list. In practice, when demand outstrips supply, other considerations prevail. Since demand for secure forensic in-patient places always outstrips supply, a chronological waiting list is never applied in practice. If the need for therapeutic security is more or less equal amongst those on a waiting list, then other considerations will determine urgency. Generally clinicians will prioritise those in prison over those who are already in a hospital elsewhere, and generally clinicians will prioritise those with the most life threatening current clinical needs over those who can safely be delayed on the waiting list or treated without admission. Further factors influencing the prioritisation of admissions include legal obligations and various pragmatic and systemic considerations concerning catchement areas and pathways through care. Systemic considerations may include contracting arrangements between public sector commissioners or insurance based funders of services and the state or independent sector providers of such services.

Lawyers may have difficulty with the concept of a non-chronological waiting list since they are accustomed to a prison system in which prison governors will invariably accept all those committed to custody by the courts, regardless of prison capacity and irrespective of the consequences for safety, over-crowding, and consequent adverse effects on the humane and therapeutic aspects of the milieu. It would not be possible to provide a hospital service on this basis, so that the purpose of committing to a hospital would be defeated if courts were given control over waiting lists or free access to hospitals irrespective of capacity or clinical need.

Further, hospitals are accustomed to managing waiting lists, whether for elective treatment or emergency treatment, employing clinical triage decision making based originally on battlefield practice in which those most in need are prioritised over those who can wait or are less needy. On this basis, the ordering of the waiting list in forensic mental health practice prioritises those with mental disorders that cannot be effectively treated or managed in prison over those with minor illnesses or simple adjustment reactions to imprisonment itself. Those who need a given level of therapeutic security are prioritised over those who need a lesser level of therapeutic security (as in court diversion schemes).

The items which follow are commonly used as a means of prioritising cases for admission, other things being equal. It is assumed that the level of the level of therapeutic security required has already been assessed, as indicated by the DUNDRUM-1 Triage Security Items.

© not to be copied or reproduced without permission.

Six items should be rated for each person on the waiting list. Because those considered for admission may be in the community, in remand or sentenced prisons or in other hospitals at higher or lower levels of therapeutic security, and because different considerations apply according to the current location, five alternative rating scales have been provided and labelled TU1A to TU1E, to indicate that only one rating should be counted for each patient.

Priority due to mental health considerations (TU2), suicide prevention (TU3) and humanitarian considerations (TU4) are each given a domain for consideration, while systemic (TU5) and legal considerations (TU6) complete the scale of items.

Some prison governors may be deterred from reforming their regimes if poor practice (e.g. prolonged seclusion, failure to provide effective protection for vulnerable prisoners) is rewarded by the transfer of challenging or vulnerable prisoners to hospital. However there will be situations where a defendant or a prisoner with an undoubted mental disorder cannot be safely managed in a prison environment.

Note that in general, those in a lower level of therapeutic security are able to benefit from medical and nursing care in a therapeutic environment, whereas those in a prison are in a non-therapeutic environment which may be toxic to their mental health. Those in prison environments therefore usually take precedence over those in hospital environments.

Validation

This scale has been validated in a prospective naturalistic observational study (Flynn et al 2011b). The items had acceptable inter-rater reliability and internal consistency. When measured at the time of going on the waiting list, the combined DUNDRUM-1 and DUNDRUM-2 score had the best area under the curve, while at the time of admission the DUNDRUM-2 score was the best predictor of admission.

© not to be copied or reproduced without permission.

TU1A: TRIAGE URGENCY: COMMUNITY FORENSIC PATIENT

This item is intended to give appropriate priority to those patients discharged from a forensic mental health service while required to comply with conditions and subject to recall.

Wherever possible it is best to preserve the working alliance and to cultivate continuity of therapeutic relationships. Much may be agreed as part of the integrated care and treatment plan, with the advance preferences of the patient playing a significant part in how intervention is staged in the event of relapse or breach of conditions. The patient may prefer to be admitted to a local catchment area service or the patient may prefer to be readmitted to the forensic service, possibly to a pre-discharge ward rather than an admission ward if appropriate. However risk management must take precedence over patient preference where there is a clear divergence between the two.

TU1A: COMMUNITY FORENSIC PATIENT

4	4.1 Is in breach of conditions of discharge **or**<br>4.2 Meets TS criteria for admission to this level of therapeutic security **and**<br>4.3 Dynamic risk factors are currently high .
3	3.1 Is relapsing or exhibiting signature signs of risk scenarios **and/or**<br>3.2 May not have breached conditions of discharge **but**<br>working alliance and risk management are better served by a readmission to the forensic service
2	2.1 Is relapsing **or**<br>2.2 Is exhibiting signature signs of risk scenarios, **or**<br>2.3 Is in breach of conditions of conditional discharge **BUT**<br>2.4 Current dynamic risk is sufficiently low to permit treatment in a lower level of therapeutic security, if necessary using the civil mental health act.
1	1.1 Essential elements of the community after-care and risk management package have broken down **but**<br>1.2 The patient is not yet relapsing, exhibiting signature signs of risk scenarios **or**<br>1.3 In breach of conditions of discharge.
0	0.1 No pre-admission assessment.

Information Quality: 0=no information; 1=staff observation only; 2=interview and staff observation; 3=family informants; 4=medical or police records.

© not to be copied or reproduced without permission.

TU1B: TRIAGE URGENCY: COURT/REMAND PRISONER

Remand prisoners have the highest psychiatric morbidity. Remand prisoners with severe mental illness may have been remanded for very minor or even nominal offences. Prison in-reach and court liaison / court diversion services exist to ensure that such persons are transferred to the appropriate community mental health facility or low secure unit at the earliest opportunity. Such patients are given a low priority for transfer to higher levels of therapeutic security, while not ruling out such a placement.

For those who have a triage security assessment indicating the need for a more secure placement, this item gives a higher weighting because placement in alternative community or lower secure places would not be appropriate. It is sometimes appropriate to consider those who have been refused bail because they are charged with a serious offence and at risk of a long sentence as if they were sentenced – see TU1C. This might lead to a rating of ‘3’ on that item rather than ‘2’ on this, and the person should be rated accordingly.

Those likely to be found unfit to stand trail (unfit to plead) should be accorded a high priority for transfer from prison to hospital so that they can be treated prior to trial. to be found unfit may result in a prolonged detention in forensic settings out of proportion to security need or risk assessment.

Those likely to be found not guilty by reason of insanity or made subject to a restriction order should similarly be given a high priority for admission, though not quite so urgently as those likely to be found unfit to stand trial. It is important that they should be treated and fully assessed prior to trial.

NB If there is no current mental disorder (broadly defined), the correct rating is zero (0), because the person is not in need of psychiatric admission or follow up.

TU1B: TRIAGE URGENCY: COURT/REMAND PRISONER

4	4.1 Severe mental illness / mental disorder **and**<br>4.2 May be found unfit to stand trial
3	3.1 Severe mental illness / mental disorder **and**<br>3.2 May be found NGRI **or**<br>3.3 Made subject to a restriction order.
2	2.1 Severe mental illness / mental disorder **and**<br>2.2 Cannot be diverted directly to a lower level of therapeutic security (includes those who are refused bail / facing a long sentence if convicted)
1	1.1 Prisoner with severe mental illness / mental disorder **but**<br>1.2 Can be diverted directly from court or from remand prison via court to a lower level of therapeutic security.
0	0.1 No pre-admission assessment **or**<br>0.2 No current evidence of mental disorder.

Information Quality: 0=no information; 1=staff observation only; 2=interview and staff observation; 3=family informants; 4=medical or police records.

© not to be copied or reproduced without permission.

TU1C: TRIAGE URGENCY: SENTENCED PRISONER

Sentenced prisoners present different issues to remand prisoners. Legally, admission to a forensic secure placement is likely to be a requirement. There are often large numbers of mentally ill persons in prison who are serving sentences and are managed by prison in-reach mental health services in much the same way they would be managed in the community. The same rights to autonomy, beneficence and confidentiality apply in prison as in the community. Those who refuse treatment must have their wishes respected, unless they lack capacity and come within the definition of mental disorder in the appropriate mental health legislation, when the legal process under the relevant mental health legislation must be followed.

It is generally not appropriate to treat without consent in a prison. Transfer to hospital may in itself be enough to alleviate a mental disorder caused by the stress of imprisonment, and the ethical principle of reciprocity holds that when depriving an individual of all or part of their autonomy or freedom due to a mental disorder, there is an obligation to supply the means of alleviating the mental disorder to restore autonomy, preventing deterioration or optimising the quality of life while subject to any form of restriction due to mental disorder. This cannot be done in prison.

Particular priority should be accorded towards the end of a sentence if a community treatment and risk management package cannot be put in place by the prison in-reach mental health team.

NB If there is no current mental disorder (broadly defined), the correct rating is zero (0), because the person is not in need of psychiatric admission or follow up.

© not to be copied or reproduced without permission.

TU1C: TRIAGE URGENCY: SENTENCED PRISONER

4	4.1 Near end of sentence, untreated **and / or**<br>4.2 With no after-care or risk management plan in place in the community **and**<br>4.3 This cannot be arranged in prison.
3	3.1 Newly ascertained mental disorder, requires assessment, treatment, through care plan and community aftercare plan **and**<br>3.2 That cannot be completed in prison.
2	2.1 Relapse of mental disorder in prison despite previous assessment, treatment, through care and aftercare plan delivered by in-reach mental health team.
1	1.1 Can be treated and maintained by prison in-reach mental health team **and**<br>2.2 after care plan can be put in place without transfer from prison to hospital.
0	0.1 No pre-admission assessment **or**<br>0.2 no current evidence of mental disorder.

Information Quality: 0=no information; 1=staff observation only; 2=interview and staff observation; 3=family informants; 4=medical or police records.

© not to be copied or reproduced without permission.

TU1D: TRIAGE URGENCY: PRIORITISING MOVES TO HIGHER LEVEL OF THERRAPEUTIC SECURITY

Transfers from a lower level of therapeutic security to a higher level may be required due to changed needs for therapeutic security per se (as assessed by the DUNDRUM-1 Triage Security items), due to an increase in assessed risk (e.g. as assessed by the HCR-20 dynamic items) or due to a specific need for specialist treatments. The ethical principles of proportionality and necessity should guide decision making. It should seldom if ever be necessary to move a patient up a level of therapeutic security only because of self harm or the prevention of suicide.

TU1D: TRIAGE URGENCY: PRIORITISING MOVES UP

NB If there is no current mental disorder (broadly defined), the correct rating is zero (0), because the person is not in need of psychiatric admission or follow up.

4	4.1 Is in another hospital and has exceeded the capacity of that hospital to safely care for the patient (e.g. may be subject to extraordinary measures see TU3) **and**<br>4.2 meets TS criteria for a move to this (higher) level of therapeutic security.
3	3.1 Is in another hospital **and**<br>3.2 meets TS criteria for a move to this (higher) level of therapeutic security (e.g. due to absconding or other TS items)
2	2.1 Is in another hospital **and**<br>2.2 meets TS criteria for a move to a level of therapeutic security intermediate between current location and this (higher) level, **but**<br>2.3 no intermediate placement is available (see also TU4).
1	1.1 Would benefit from a move to a higher level of therapeutic security in order to engage with specialist treatment programmes
0	0.1 No pre-admission assessment **or**<br>0.2 no current evidence of mental disorder **or**<br>0.3 is not in another hospital

Information Quality: 0=no information; 1=staff observation only; 2=interview and staff observation; 3=family informants; 4=medical or police records.

© not to be copied or reproduced without permission.

TU1E: TRIAGE URGENCY: PRIORITISING MOVES TO SAME OR LOWER LEVEL OF THERAPEUTIC SECURITY

Patients have a right to be detained in no greater a degree of therapeutic security than is necessary and proportionate to their need. The DUNDRUM-1 TS items are a guide to this need. Risk assessment e.g. with the HCR-20 is complimentary to and adds to such an assessment. The DUNDRUM-3 Recovery and DUNDRUM-4 Programme Completion items are also a useful guide to readiness for moves to lower levels of therapeutic security. Moves from acute low secure (PICU) to longer term low secure units, or from medium term to longer term medium secure units may also be appropriate when progress in treatment is unlikely to lead to a move to a lower level of therapeutic security and in addition the quality of life is enhanced by such a move.

TU1E: TRIAGE URGENCY: PRIORITISING MOVES TO SAME OR LOWER LEVEL OF THERAPEUTIC SECURITY

NB If there is no current mental disorder (broadly defined), the correct rating is zero (0), because the person is not in need of psychiatric admission or follow up.

4	4.1 Is in another hospital at a higher level of therapeutic security **and**<br>4.2 would benefit from a move to a lower level of therapeutic security (at this hospital) **OR**<br>4.3 would benefit from a move to this specialised service at the same level of therapeutic security e.g forensic intellectual disability service, acquired brain injury service.
3	3.1 Is in another hospital (which may be out of catchment area) at the same or higher level of therapeutic security **and**<br>3.2 requires admission to this unit (at the same level of therapeutic security or a lower level) to connect with a pathway through care locally.
2	2.1 Is in another hospital (which may be out of catchment area) at the same or higher level of therapeutic security **and**<br>2.2 would benefit from a move to this hospital (at the same or lower level) to engage in rehabilitation or family therapy programmes not available at the current placement (e.g. family live near this place), **OR**<br>2.3 would benefit from a move to this hospital (at the same or lower level) to have a better quality of life for longer term care at the same level of therapeutic security
1	1.1 Is in an out of catchment area hospital at the same level of therapeutic security **and**<br>1.2 would benefit from a move to a hospital in the catchment area nearer to family and own community.
0	0.1 No pre-admission assessment **or**<br>0.2 no current evidence of mental disorder **or**<br>0.3 is not in another hospital.

Information Quality: 0=no information; 1=staff observation only; 2=interview and staff observation; 3=family informants; 4=medical or police records.

© not to be copied or reproduced without permission.

TU2: TRIAGE URGENCY: MENTAL HEALTH

This item gives weight to clinical urgency, with life threatening problems taking precedence. Physical illness alone will not require admission to a therapeutically secure mental health unit and is better dealt with in a general hospital. In a general hospital security staff from the prison may be allocated to stay with the person or if the patient is already in a mental health service, staff from the mental health unit may be present by the bedside.

NB If there is no current mental disorder (broadly defined), the correct rating is zero (0), because the person is not in need of psychiatric admission or follow up.

TU2: TRIAGE URGENCY: MENTAL HEALTH

4	A life-threatening state e.g. catatonic stupor **or** acute excited state that cannot be managed<br>4.1 in current hospital at lesser level of therapeutic security **or**<br>4.2 in prison
3	3.1 Deteriorating mental state (psychosis) **and**<br>3.2 deteriorating physical state **either**<br>3.3 in prison **or**<br>3.4 in current hospital placement due to lack of therapeutic security
2	2.1 Stable but unsatisfactory mental health **and**<br>2.2 cannot be treated for severe mental illness in present placement **either**<br>2.3 in a lower level of therapeutic security **or**<br>2.4 in prison - e.g. in prison requires transfer under Mental Health legislation for treatment without consent.
1	1.1 Accepting treatment for severe mental illness in present place, whether in community, hospital or prison **but**<br>1.2 would respond better or would benefit to a greater degree if transferred to hospital at this level of therapeutic security.
0	0.1 No pre-admission assessment **or**<br>0.2 no current evidence of mental disorder.

Information Quality: 0=no information; 1=staff observation only; 2=interview and staff observation; 3=family informants; 4=medical or police records.

© not to be copied or reproduced without permission.

TU3: TRIAGE URGENCY: SUICIDE PREVENTION

This item is intended to give appropriate weight to those who need admission to hospital in order to manage the risk of suicide. An assessment that took account of risk (probability) only would prioritise many who engage in repetitive self harming behaviour that is of low lethality. The S-RAMM (Bouch & Marshall 2003, Ijaz et al 2009, Fagan et al 2009) identifies those who have preferred methods which are of high lethality. This item relies on the dichotomy between probability or immediacy on the one hand, and gravity (lethality) on the other. This emphasises the sensitivity of the item to change over time.

Traditionally, those remanded in custody charged with murder or rape were regarded as at high risk of completed suicide, particularly where the scenario is of a failed extended suicide. Failed ‘suicide by cop’ may also be a high risk. Brophy (2003) has shown that those charged with sex offences are at high risk of suicide, particularly those charged with offences against children. The same paper indicated however that the risk was higher for those still in the community, with those remanded in custody at no higher risk than other prisoners.

In general those who are already in hospital at any level of therapeutic security can be cared for sufficiently to prevent suicide e.g. by close nursing observations and detention under civil mental health legislation, though occasionally a high absconding risk may require admission to a low secure unit. Accordingly those already in a hospital are ‘capped’ at a rating of ‘2’ for this item.

© not to be copied or reproduced without permission.

TU3: TRIAGE URGENCY: SUICIDE PREVENTION

NB If there is no current mental disorder (broadly defined), the correct rating is zero (0), because the person is not in need of psychiatric admission or follow up.

4	4.1 recent high lethality suicide attempt, **and**<br>4.2 is in prison **and**<br>4.3 Dynamic risk factors high currently (e.g. recent failed extended suicide or suicide by cop, or stigmatising offence, or TAG assessment).
3	3.1 High lethality attempts **but**<br>3.2 not recent **or**<br>3.3 not high risk currently (low dynamic risk) while in prison.
2	2.1 High risk of low-lethality self-harm **or**<br>2.2 is already in any hospital placement.
1	1.1 Low risk currently **and**<br>1.2 low-lethality behaviours.
0	0.1 No pre-admission assessment **or**<br>0.2 no current evidence of mental disorder **or**<br>0.3 no suicide risk / behaviour.

Information Quality: 0=no information; 1=staff observation only; 2=interview and staff observation; 3=family informants; 4=medical or police records.

© not to be copied or reproduced without permission.

TU4 TRIAGE URGENCY: HUMANITARIAN

This item gives weight to humanitarian and human rights considerations. It is essential to avoid having to impose conditions of treatment or detention that might constitute cruel, unusual or inhuman treatment. If oppressive measures such as physical restraint, seclusion or any other form of coercion are used due to a mental disorder, and if transfer to a therapeutically secure hospital would allow care or treatment without these measures then the transfer should be prioritised accordingly.

Note that in hospital seclusion, restraint and other extraordinary measures may be avoided or minimised by enhanced nursing observations including 2 to 1 nursing and the use of higher staff to patient ratios generally.

NB If there is no current mental disorder (broadly defined), the correct rating is zero (0), because the person is not in need of psychiatric admission or follow up.

TU4 TRIAGE URGENCY: HUMANITARIAN

4	4.1 Is endangering self and others in present place despite extra-ordinary measures e.g. prolonged seclusion or restraint, **and**<br>4.2 is in prison (see TU1B or TU1C)
3	3.1 Requires extra-ordinary means in present placement e.g, prolonged seclusion or restraint with no prospect of improvement **and**<br>3.2 is in prison (see TU1B or TU1C)
2	2.1 Is endangering self and others in present place despite extra-ordinary measures e.g. prolonged seclusion or restraint, **but**<br>2.2 is currently in a hospital (see TU1D)
1	1.1 Requires extra-ordinary means in present placement e.g. prolonged seclusion or restraint with no prospect of improvement **and**<br>1.2 is in hospital (see TU1D).
0	0.1 No pre-admission assessment **or**<br>0.2 no current evidence of mental disorder **or**<br>0.3 no necessity to admit **or**<br>0.4 can be managed with precautions but without extra-ordinary means e.g. in a shared cell, with enhanced observation levels.

Information Quality: 0=no information; 1=staff observation only; 2=interview and staff observation; 3=family informants; 4=medical or police records.

© not to be copied or reproduced without permission.

TU 5 TRIAGE URGENCY: SYSTEMIC

This item assesses the extent to which it is systemically appropriate within an overall mental health service for a population to consider the patient for the level of security provided by this service. A pragmatic, patient centred ‘best interests’ approach must at all times take precedence over other considerations. This is particularly true when catchment area and resource issues are at play in a public health service. As a guide to the appropriateness of admission to a given level of therapeutic security, the DUNDRUM-1 Triage Security rating items and scale can be used.

The distinction made here between ‘soft’ obstacles to admission and ‘hard’ resource issues is an example of pragmatic decision making. Yielding too readily to ‘soft’ obstacles however is systemically dysfunctional and leads to ‘system drift’ whereby appropriately resourced services decline to offer the service for which they have been commissioned and resourced. These issues should wherever possible be resolved by recourse to the DUNDRUM-1 Security items on a case by case basis and as part of a systems audit.

This item may be seen as an additional weighting for issues dealt with in various parts of TU1.

© not to be copied or reproduced without permission.

TU 5 TRIAGE URGENCY: SYSTEMIC

NB If there is no current mental disorder (LEGALLY defined), the correct rating is zero (0), because the person is not in need of psychiatric admission or follow up.

4	4.1 Due to assessed triage security needs, it is appropriate to admit the patient to this level of therapeutic security (not to a lower level) **and**<br>4.2 this is the catchment area service.
3	3.1 Due to assessed triage security needs it is appropriate to admit the patient to a lower level of therapeutic security **but**<br>3.2 It is necessary to admit the patient to this level of therapeutic security a lower level, though appropriate is not available anywhere in the jurisdiction due to resource constraints.
2	2.1 Due to assessed triage security needs it is appropriate to admit the patient to a lower level of therapeutic security **but**<br>2.2 It is necessary to admit the patient to this level of therapeutic security a lower level, though appropriate is not available <u>in the catchment area</u> due to resource constraints.<br>NB the more appropriate lower level of therapeutic security should be sought in other catchment areas (see TU1E).
1	1.1 Due to assessed triage security needs it is appropriate to admit the patient to a lower level of therapeutic security **but**<br>1.2 It is necessary to admit the patient to this level of therapeutic security because lower levels though appropriate are not accessible for ‘soft’ reasons e.g. due to catchment area disagreements or local stigma.
0	No mental disorder OR higher levels of therapeutic security are not available.

© not to be copied or reproduced without permission.

TU6 TRIAGE URGENCY: LEGAL URGENCY

These items give rise to greater conceptual difficulty than any other in this structured professional judgement instrument. All other items reflect the ethical obligation to put the best interests of the person first and to ensure that the appropriate safe therapeutic environment is used to enable the recovery and return to autonomy of the person concerned. This item however prioritises different principles – legal procedures rather than consequences, liberty (in a legal sense) rather than recovery, and where conflicts arise they are often the result of lack of clarification or communication of these issues. N.B. clinical decision makers are advised to seek legal advice as a matter of urgency whenever any difficulty arises in relation to such matters.

It is the view of the authors that legal orders causing the admission of a person who is before the courts in preference to a more medically needy person as rated in these items, particularly DUNDRUM-1 and DUNDRUM-2 are always wrong in principle and in practice. It is the responsibility of the clinicians to ensure that the legal authority making such orders should be aware of the probable consequences of their actions particularly the consequences for those who are for clinical reasons in greater, more urgent need of the hospital bed. There is an inherent injustice when decisions are made deliberately blind to the consequences for others. There is also an inherent error when the responsible decisions normally vested by society in doctors are instead taken by lawyers who are exempt from responsibility for the consequences.

The rating system below prioritises this principle of continuity of responsibility – a decision regarding urgency is more weighty if made by the admitting institution than when made by an expert who carries no clinical responsibility for the consequences.

© not to be copied or reproduced without permission.

TU6 TRIAGE URGENCY: LEGAL URGENCY

NB If there is no current mental disorder (LEGALLY defined), the correct rating is zero (0), because the person is not in need of psychiatric admission or follow up.

4	4.1 A ‘forthwith’ order has been made arising from judicial review or habeas corpus proceedings in connection with detention in prison or elsewhere while awaiting a hospital place **or**<br>4.2 an order has been correctly completed by a court obliging an admission at once e.g. unfit to plead or NGRI **or**<br>4.3 a recall order for a conditionally discharged patient has been made and requires admission to this hospital at once **or**<br>4.4 an order has been made for prison to hospital transfer with immediate effect.
3	3.1 A court order or Mental Health Tribunal order has been made to admit within a defined time period e.g. one or two weeks **or**<br>3.2 a court order to admit forthwith (JR, Unfit, NGRI) scheduled and likely to be made within the next week **or**<br>3.3 an order has been made for prison to hospital transfer within a defined time period e.g. one or two weeks
2	2.1 Judicial review or similar proceedings (fitness to stand trial, NGRI, hospital order or restriction order) initiated with a view to admission and likely to succeed **or**<br>2.2 an order for prison to hospital transfer may be made, subject to bed availability and triage considerations
1	1.1 ‘Request’ from any court for a medico-legal report, or for advice or assistance regarding hospital admission – NB an alternative disposal may be more appropriate, see TS items. **or**<br>1.2 ‘Approval’ for admission or transfer by a Mental Health Tribunal.
0	0.1 No court order, **or**<br>0.2 ‘Order’ to admit by a court that lacks statutory power or inherent powers of High Court (i.e. power to make such an order), **or**<br>0.3 any order that on its face is invalid. NB seek legal advice at once.

Information Quality: 0=no information; 1=staff observation only; 2=interview and staff observation; 3=family informants; 4=medical or police records.

© not to be copied or reproduced without permission.

DUNDRUM-3: Programme Completion Items:

For those discharged from a specialised forensic mental health service or moved from a higher to a lower level of therapeutic security, it is reasonable to expect that they would have completed programmes relevant to the risk items that required their original admission to the service. There should be a relationship between completion of the stages of these treatment programmes and progress from admission / high secure units to medium secure and on to rehabilitation and recovery (minimum security, pre-discharge) units and community follow-up. In devising this structured professional judgement instrument and the companion DUNDRUM-4 recovery items, we have been greatly influenced by the concept underpinning the HCR-20 Risk Management Manual (Douglas et al 2001). In practice we believe the items in the DUNDRUM-3 Programme Completion instrument will consistently address the risk factors identified in the course of risk assessment as well as in the assessed need for security.

Our starting point has been the proposition that remission of symptoms is not the same as recovery (Andreasen et al 2005) Recovery can be described in terms of stages and processes (Andresen et al 2003, Weeks et al 2010).

Five Pillars of Treatment
The programme completion items reflect the organisation of treatment programmes in practice, according to five ‘pillars’ of treatment: physical health, mental health, drugs and alcohol recovery, problem behaviours (offence related behaviour) and a fifth broad category that includes social, family and occupational life.

Taken together these five pillars are intended to cover the domains of health defined by the WHO (1946) “*Health is a state of complete physical, mental and social well-being and not merely the absence of disease or infirmity*”. A more recent definition from the WHO (1986), The Ottawa Charter for Health Promotion holds that health is *"a resource for everyday life, not the objective of living. Health is a positive concept emphasizing social and personal resources, as well as physical capacities."* (see also Jadad & O’Grady 2008).

There are existing research instruments and clinical rating scales that cover similar issues, often in the context of needs assessment research in forensic settings (Cohen & Eastman 2000). The TAPS project used the social behaviour schedule (Wykes et al 1986) and this has been used as part of needs assessment in forensic populations in different jurisdictions (Pierzchniak et al 1999, O’Neill et al 2003). We have shown (Pillay et al 2008) that the more recent research and clinical instruments for assessing treatment need such as the CANFOR (Thomas et al 2003) and HoNOS-SECURE (Sugarman & Walker 2004) appear to reflect differences in levels of met and unmet need for patients at different levels of therapeutic security – admission/high secure units, medium secure units and rehabilitation and pre-discharge units. Although these scales did differ significantly as patients progressed, the differences were small in absolute terms and confidence intervals overlapped.

© not to be copied or reproduced without permission.

There is good evidence that the HCR-20 clinical items demonstrate a similar pattern of stratification along the recovery pathway (Dernevik et al 2002, Muller-Isberner et al 2007) along with measures of mental state and global function (Pillay et al 2008) such as the PANSS (Kay et al 1987) and GAF (American Psychiatric Association 1994). Again we recommend that the DUNDRUM-3 & 4 items should be used with the HCR-20 or other risk assessment instruments. These scales measure something complimentary to risk and are not intended as risk assessments.

We have collated the content of existing scales such as the CANFOR and HoNOS and based on our experience of them, added items that we believe are relevant to the relationship between treatment, recovery and changing security need.

Item ratings and theory: Maslow, cycle of change and engagement

The rating scales for the recovery items include elements of Maslow's (1943) hierarchy of needs and motivation. The programme completion stages referred to at level '4' are mostly sufficient for physiological needs at best. Level '3' should have elements of safety concerning the basics of life. Level '2' concerns friendship and family relationships. Level '1' aspires to self-esteem, confidence and social standing. Level '0' emphasises the additional aspects of self-actualisation – morality, creativity, problem solving, acceptance of facts. While modern theorists tend towards the view that these needs are universal rather than hierarchical, the progression from need for basic care to autonomy fits well with the recovery model.

These rating items also include elements of the trans-theoretical model or stages of change (Prochaska & DiClemente 1983, DiClemente et al 1991) organised into five stages, starting with pre-contemplation (rated '4'), contemplation, preparation (rated '3'), action (rated '2'), maintenance (rated '1' or '0'), with motivational work concerning ambivalence and decisional balance.

'Engagement' should be demonstrated through more than simply having attended all sessions of a programme. Engagement should include evidence that the person has benefitted from the programme. Evidence of engagement and benefit at its most basic would include passive participation, at the next level would include evidence of active engagement with retained information, changed attitudes and altered behaviour. Evidence of positive engagement includes showing the ability to personalise the content by giving examples of one's own experiences relating to her/himself that are relevant to the content of the programme. Successful completion should mean having attended at least 90% of scheduled sessions in a programme during which the patient has actively participated. Those delivering programmes must therefore have time to complete reports on programme completion and there must be some system for outcome assessment.

Recovery can be described as five stages (Andresen et al 2003, Weeks et al 2010) – 'moratorium' a stage of hopelessness and self-protective withdrawal; awareness, the realisation that recovery and a fulfilling life is possible; preparation – the search for personal resources and external sources of help; rebuilding – taking positive steps towards meaningful goals; and growth – a sense of control over one's life and looking

© not to be copied or reproduced without permission.

forward to the future. In general terms, the transitions from one stage to the next are mediated by four processes – finding hope, taking responsibility, establishing a positive identity and finding meaning and purpose in life.

Where the rating scales for individual items refer to well-known programmes such as the Wellness Recovery Action Programme (WRAP), these are intended only as examples. Any similar programme would do. It is of course better to use a 'manualised' programme - a course of therapy that has been written in the form of a curriculum over a defined number of sessions, with learning goals for each session and pre-defined outcome measures. It is also best to use a treatment programme that has been validated, at least by change of outcome measures and preferably by demonstrating change in real-world outcomes such as reduced re-admission or re-offending. The use of a 'manualised' programme and appropriate training for the therapists ensures fidelity to the treatment programme as it was validated. However at present there is very little formal validation available for such programmes – this should be a topic for future development.

In general, if there is no problem of the sort referred to, a '0' rating will apply e.g. for P2 Drugs and Alcohol where there is no history of any such problem.

Like the recovery items, those who are mostly rated '4' are probably unlikely to be ready for a move to a medium secure setting, or to any setting at a lower level of security than their current placement; those mostly rated '3' may be ready for a move from a high secure to a medium secure setting; those mostly rated '2' may be ready for a move from medium to low security; those mostly rated '1' may be ready for a move to an open or community placement – though the availability of a high level of community support, structure and supervision, mandated if necessary by legally binding conditional discharge with a power of recall, may be a part of such a decision. Finally, those rated '0' in a range of areas relevant to their risk assessment may be ready for an absolute legal discharge though this should be an individualised decision in all cases.

© not to be copied or reproduced without permission.

	Cycle of change (Prochaska & DiClement 1983)	Engagement	Recovery (Andresen et al 2003)	Maslow (1943)
O: ready for independence	Maintenance, stability	Taking personal responsibility	Growth	Self-actualisation
1: ready for a move to supported community living e.g. conditional discharge or community treatment order	Maintenance, supported	Positive engagement	Rebuilding	Self-esteem, confidence, social standing
2: ready for a move e.g. from medium to low security	Action / decisional balance	Active engagement	Preparation	Friendship and family relationships
3: ready for a move e.g. from high to medium security	Contemplation & preparation, ambivalence	Passive engagement	Awareness	Safety and basics of life
4: not ready to move down a level of security	Pre-contemplation.	Reluctance / resistance	Moratorium	Physiological needs

Validity

The DUNDRUM-3 and DUNDRUM-4 have been shown to have excellent internal consistency and inter-rater reliability (O'Dwyer et al 2011).

For forensic in-patients with severe mental illnesses in a forensic hospital, those who had positive moves (from more secure to less secure units) had lower (better) mean scores for the DUNDRUM-1, DUNDRUM-3 and DUNDRUM-4 when adjusted for location, but adjusting for risk (HCR-20) eliminated this difference for the DUNDRUM-3 and DUNDRUM-4. The DUNDRUM-3 and DUNDRUM-4 scores were higher (worse) for those who moved from less secure to more secure units (negative moves) when adjusted for location at baseline. Location at baseline, with the DUNDRUM-1 and HCR-20 dynamic scores were more robust predictors of positive and negative moves in all analyses. The DUNDRUM-3 and DUNDRUM-4 measures were not independent of risk as measured by the HCR-20 dynamic score but they appear to measure something complimentary to risk (Davoren et al 2013a).

© not to be copied or reproduced without permission.

The DUNDRUM-3 and DUNDRUM-4 emerged as the best predictors of conditional discharge by the statutory review board. The DUNDRUM-3 distinguished which patients were subsequently conditionally discharged by the Mental Health Review Board (AUC=0.902, p<0.001) as did the DUNDRUM-4 (AUC=0.848, p<0.001). Item to outcome analysis showed each item of both scales performed significantly better than chance. THE HCR-20 also distinguished those later discharged (AUC=0.838, p<0.001) as did other measures of risk and protection (S-RAMM, SAPROF, START, PANSS, GAF). The DUNDRUM-3 and DUNDRUM-4 scores remained significantly lower (better) for those conditionally discharged even when corrected for the HCR-20 total score. Item to outcome analyses and logistic regression analyses showed that the strongest antecedents of discharge were the GAF and DUNDRUM-3 programme completion scores (Davoren et al 2013b).

© not to be copied or reproduced without permission.

Programme Completion Item P1: Physical Health:

This item rates the patient's progress in actively managing their physical health. A preliminary step for most would be an education programme regarding physical health. This would be followed by a programme specifically focusing on physical health and recovery. While the scoring items refer to particular manualised programmes such as Solutions for Wellness, other programmes could as easily be substituted. For physical health, the emphasis has to be on having regular patterns of self-care including exercise, diet, sleep and engagement with clinics providing for any specific physical needs such as diabetes, cholesterol monitoring or other physical problems including regular health checks and national screening programmes

© not to be copied or reproduced without permission.

Coding: P1. Physical Health

4	4.1 Has not yet successfully completed any programme concerning physical health awareness **or**<br>4.2 Does not choose healthy physical lifestyle options despite staff encouragement **or**<br>4.3 Is dependent on nursing care for many basic activities of daily living and self care concerning physical health.
3	3.1 As a minimum has successfully completed a primary health care assessment and follow-up programme **and**<br>3.2 Takes a passive interest (contemplates, prepares for action) in balancing diet and exercise.<br>3.3 **But** only engages with healthy lifestyle options when prompted by staff to do so.
2	2.1 As a minimum has successfully completed education programmes regarding physical health (e.g. 'Solutions for Wellness' or similar programme) **and**<br>2.2 Evidence of change is sustained over time – at least twelve months e.g **and**<br>2.3 Shows active interest in preparing healthy meals and/or takes exercise regularly, enjoys some form of sport or exercise.<br>2.4 **May** sometimes need prompting to adhere to physical health management programmes.
1	1.1 Is self-medicating and self-caring for physical health and actively engaged with follow-up / maintenance programmes for physical health as appropriate e.g. self-monitors blood sugar if diabetic **and**<br>1.2 Has a regular dietary and exercise pattern and routine, has incorporated healthy eating and exercise programme into daily routine **and**<br>1.3 with minimum prompting takes care of own appearance and health as a source of self-esteem and dignity.
0	For a period of five years -<br>0.1 Has taken responsibility for own active recovery and personal physical health **and**<br>0.2 Has a regular pattern of self-care and self-medication for physical health **and**<br>0.3 Self-presentation to primary care as appropriate.

Information Quality: 0=no information; 1=staff observation only; 2=interview and staff observation; 3=family informants; 4=medical or police records.

"successfully completed" means has attended at least 90% of scheduled sessions in a programme during which the patient engaged fully, has actively participated and has shown the ability to personalise the content by giving examples from own experience relating to him/herself.

"Engaged" means enters into and commits to, as shown by consistency and initiative, effort and supportiveness of the goals of an activity or programme.

© not to be copied or reproduced without permission.

Programme Completion Item P2: Mental Health:

This item rates the patient's progress in actively managing their mental health. A preliminary step for most would be an education programme regarding physical and mental health. This would be followed by a programme specifically focusing on mental health and recovery. While the scoring items refer to particular manualised programmes such as Wellness Recovery Action Programme (WRAP), other programmes could as easily be substituted.

Remission as defined by the *Remission in Schizophrenia Working Group* (Andreasen et al 2005) and for depression (Frank et al 1991) is not an essential, though it is a desirable goal. That is not the focus of this item.

© not to be copied or reproduced without permission.

Coding: P2. Mental Health

4	4.1 Has not yet successfully completed any programme concerning mental illness / mental health awareness **or**<br>4.2 Requires supervised medication e.g. depot neuroleptic, observation swallowing meds, regular blood level checks.
3	3.2 As a minimum has successfully completed a 'Wellness programme' or equivalent **and**<br>3.3 Shows interest (contemplation / preparation) in learning about mental health and engages in programmes for relapse prevention. **May** need encouragement.<br>3.4 **May** still need supervision of compliance with medication for mental health needs.
2	2.1 As a minimum has successfully completed a 'Wellness Recovery Action Plan' education programme **and**<br>2.2 Evidence of change in relation to mental health awareness is sustained over time – at least twelve months **and**<br>2.3 Takes an active interest in balancing use of time between work (broadly defined), family and friends, leisure and creativity.<br>2.3 May need some prompting from staff and carers concerning mental health needs.
1	1.1 Should be self-medicating and self-caring for mental health **and**<br>1.2 fully engaged with follow-up / maintenance programmes for mental health e.g. maintains contact with mentors and/or advocates (where available) as well as mental health professionals **and**<br>1.3 Has a regular pattern and routine of activities over the day, week and year **and**<br>1.4 Derives satisfaction from successful mental health achievements.
0	For a period of five years<br>0.1 should have maintained an interest in active recovery and personal mental health, including (**and)**<br>0.2 A regular pattern of self-care and self-medication for mental health **and**<br>0.3 **when/if** necessary **then** self-presentation to mental health team e.g. keeps appointments, recognises early signs of relapse and self-presents.

Information Quality: 0=no information; 1=staff observation only; 2=interview and staff observation; 3=family informants; 4=medical or police records.

"successfully completed" means has attended at least 90% of scheduled sessions in a programme during which the patient engaged fully, has actively participated and has shown the ability to personalise the content by giving examples from own experience relating to him/herself.

"Engaged" means enters into and commits to, as shown by consistency and initiative, effort and supportiveness of the goals of an activity or programme.

© not to be copied or reproduced without permission.

Programme Completion Item P3: Drugs and Alcohol:

Because the clientele of a therapeutically secure mental health service is selected for severe mental illness, but the majority will have co-morbid substance misuse problems, and because the evidence that it is the combination of severe mental illness and intoxication that most predisposes to violence, the emphasis in forensic mental health services is on abstinence. The evidence for a sustained return to controlled drinking after a period of dependence is poor, and would not necessarily assist recovery from mental illness or reduce the risk of violence.

The aim is for the patient to participate fully in a graded series of programmes, starting with an education programme, progressing to an abstinence oriented recovery programme and followed by a maintenance / top-up programme.

© not to be copied or reproduced without permission.

Coding: P3. Drugs and Alcohol

4	If relevant<br>4.1 Pre-contemplation Has not yet successfully completed any programme concerning substance misuse.
3	If relevant<br>3.1 Contemplation or ambivalence. As a minimum has successfully completed an education programme regarding drugs and alcohol.
2	If relevant<br>2.1 As a minimum has successfully completed a full drugs and alcohol recovery programme **and**<br>2.2 Is working towards abstinence (action) e.g. by limiting/ending contact with former circle of users **and**<br>2.3 Evidence of change is sustained over time – at least twelve months e.g sustained abstinence.<br>2.4 **But** may need continued prompting / guidance.
1	If relevant<br>1.1 Should be fully engaged with drugs and alcohol recovery follow-up / maintenance programmes as appropriate **and**<br>1.2 Random screening is consistently negative.<br>1.3 Where (**if**) 'slips' have occurred, (**then**) copes by seeking help.<br>1.4 Has regular patterns and routines in these domains **and**<br>1.5 Derives self-confidence from identity as an abstinent person in recovery.
0	If relevant, for a period of five years -<br>0.1 should have maintained an interest in active recovery with total abstinence for those with a history of substance misuse or dependence.

Information Quality: 0=no information; 1=staff observation only; 2=interview and staff observation; 3=family informants; 4=medical or police records.

“successfully completed” means has attended at least 90% of scheduled sessions in a programme during which the patient engaged fully, has actively participated and has shown the ability to personalise the content by giving examples from own experience relating to him/herself.

“Engaged” means enters into and commits to, as shown by consistency and initiative, effort and supportiveness of the goals of an activity or programme.

© not to be copied or reproduced without permission.

Programme Completion Item P4: Problem Behaviours:

The expectation is that a preliminary stage of treatment would be fairly general consisting of enhanced thinking skills (ETS) and a selection of modules resembling dialectic behaviour therapy (a ‘balance’ programme). More specific programmes should follow, such as anger management (or CALM), healthy sexual functioning (or sex offender treatment programmes), victim impact and empathy programmes (including restorative programmes where possible) or full DBT programmes. Individual work should accompany such programmes. A primary goal should be to complete a Five WH programme (who, what, where, when, why) based on working through the book of evidence / witness statements presented at trial. Similar 'ABC' (antecedent, behaviour, consequences) approaches may be taken for problem behaviours in hospitals or other institutions. Individual work may also include grief work, cognitive work for depression and cognitive work regarding the index offence or behaviour.

© not to be copied or reproduced without permission.

Coding: P4. Problem Behaviours

4	4.1 Has not yet successfully completed any programme concerning offence related behaviour **or**.<br>4.2 Psychological / interpersonal aspects of offence related behaviour specific to the person are still in evidence **or**<br>4.3 the patient is not yet contemplating change in relation to offending behaviour.
3	3.1 As a minimum has successfully completed any general programmes concerning patterns underlying high risk behaviours such as meta-cognitive training, enhanced thinking skills or 'balance' programme (DBT modules) **and**<br>3.2 Patient accepts the need for change (contemplation/preparation) in psychological or interpersonal style specific to offending behaviour.
2	2.1 As a minimum has successfully completed offence related programmes e.g. anger management, healthy relationships and healthy sexual functioning, '5 WH' work, as individually appropriate **and**<br>2.2 Evidence of change is sustained over time – at least twelve months e.g. not requiring de-escalation.
1	1.1 Should be engaged with a well-balanced and regular daily and weekly programme of self-care, occupation and leisure **and**.<br>1.2 **If** there have been behavioural 'slips' or new stresses **then** copes by seeking appropriate help from the team in a timely way **and**<br>1.3 Derives confidence and self-esteem from changes associated with avoiding problem behaviours.
0	For a period of five years -<br>0.1 should have had no offending behaviour or high risk behaviours for offending both specific to the patient and general **and**<br>0.2 Espouses pro-social beliefs, renounces pro-criminal beliefs.

Information Quality: 0=no information; 1=staff observation only; 2=interview and staff observation; 3=family informants; 4=medical or police records.

"successfully completed" means has attended at least 90% of scheduled sessions in a programme during which the patient engaged fully, has actively participated and has shown the ability to personalise the content by giving examples from own experience relating to him/herself.

"Engaged" means enters into and commits to, as shown by consistency and initiative, effort and supportiveness of the goals of an activity or programme.

© not to be copied or reproduced without permission.

Programme Completion Item P5: Self Care and Activities of Daily Living:

,

The progression towards recovery here is likely to start with a basic course in kitchen hygiene and safety. An assessment such as the AMPS may underpin the programme that follows. Self-catering, including budgeting skills, shopping and use of public transport might usefully follow and give a purpose to the progressive use of leave as described in R4. Useful tools established in this domain include the Behavioural Status Index (BSI Reed et al 2000)

The aim is to achieve a well-balanced working week and a balanced life-style, in keeping with MOHO principles.

© not to be copied or reproduced without permission.

Coding: P5. Self-Care and Activities of Daily Living

4	4.1 Has not yet completed any programme concerning self-care or basic social skills, activities of daily living or interaction with others on the ward **or**<br>4.2 Appears institutionalised / dependent over and above negative symptoms.
3	3.1 As a minimum has successfully completed assessments of abilities (AMPS, MOHO) **and**.<br>3.2 Shows a passive interest in aspects of self-care and activities of daily living (contemplation – preparation).
2	For at least twelve months -<br>2.1 As a minimum has successfully completed OT courses on self-catering, budgeting, shopping, use of public transport **and**.<br>2.2 should be safe in workshops with shadow-boarded tools **or**<br>2.3 should be safe in kitchen-based groups.<br>2.4 May need prompting.
1	1.1 Is self-caring (cooking, laundry, shopping, budgeting) and fully engaged with follow-up / maintenance programmes as appropriate **and**<br>1.2 Is engaged with a well-balanced daily and weekly programme of self-care **and**<br>1.3 Takes pride in / derives self-confidence from self-care and dignity.
0	For a period of five years -<br>0.1 has maintained an interest in active recovery and personal mental and physical health, including (**and**)<br>0.2 A regular pattern of self-care (cooking, laundry, shopping, budgeting independently).

Information Quality: 0=no information; 1=staff observation only; 2=interview and staff observation; 3=family informants; 4=medical or police records.

“successfully completed” means has attended at least 90% of scheduled sessions in a programme during which the patient engaged fully, has actively participated and has shown the ability to personalise the content by giving examples from own experience relating to him/herself.

“Engaged” means enters into and commits to, as shown by consistency and initiative, effort and supportiveness of the goals of an activity or programme.

© not to be copied or reproduced without permission.

Programme Completion Item P6: Education, Occupation and Creativity

This refers to some of the elements regarded by Maslow as essential for self-actualisation. However aspects of these activities should be present for all pillars/domains. The progression here is from basic literacy, numeracy and communication skills to increasing engagement with occupational and leisure activities. Sport, awareness of current affairs and creative activities are considered broadly equivalent.

© not to be copied or reproduced without permission.

Coding: P6 Education, Occupation, Creativity

4	4.1 Has not yet engaged in any programme concerning literacy or study skills, occupations or creativity **or**<br>4.2 May need direction or structuring to attend any such activities.
3	3.1 As a minimum has shown passive interest (contemplation – preparation) in any programme concerning literacy or study skills, occupations or creativity.
2	2.1 As a minimum has successfully participated in programmes covering education and/or occupational skills and routines, and/or some creative activities (film club, creative writing, music, art, performance) and/or current affairs awareness **and**<br>2.2 Evidence of change/commitment to these activities is sustained over time – at least twelve months.<br>2.3 May need some prompting.
1	1.1 Should be engaged with a well-balanced regular daily and weekly programme of occupation and leisure **and**<br>1.2 Some sport, creative or social / current affairs activities should be included **and**<br>1.3 Derives personal satisfaction from these activities and identifies with them.
0	For a period of five years -<br>0.1 has maintained a regular pattern of education or occupation and leisure **and**<br>0.2 Has a range of interests and activities including education and/or work (paid or un-paid) and/or sport and/or creativity and/or awareness of current affairs

Information Quality: 0=no information; 1=staff observation only; 2=interview and staff observation; 3=family informants; 4=medical or police records.

“successfully completed” means has attended at least 90% of scheduled sessions in a programme during which the patient engaged fully, has actively participated and has shown the ability to personalise the content by giving examples from own experience relating to him/herself.

“Engaged” means enters into and commits to, as shown by consistency and initiative, effort and supportiveness of the goals of an activity or programme.

© not to be copied or reproduced without permission.

Programme Completion Item P7: Family and Social Networks: Friendship and Intimacy

The model here is a progression from quiet co-existence with fellow-patients through sustaining friendship without repetitive conflict to extending this style of relating to family and friends in the community. Formal family therapy may be an individualised part of this domain. However the successful management of relational therapeutic security, and in particular that aspect described as qualitative relational security emphasises the role of the nurses and other MDT members in recognising dysfunction in the ward based milieu of relationships and finding ways to address this.

© not to be copied or reproduced without permission.

Coding: P7 Family and Social Networks, Friendship and Intimacy

4	4.1 Has no interest in interaction with fellow patients, staff **or**<br>4.2 has no interest in interaction with friends or relatives in the community **or**<br>4.3 Has a pattern of dysfunctional or conflicting interactions and relationships in hospital or in the community.
3	3.1 As a minimum has a regular pattern of neutral or friendly interactions with staff **and** fellow-patients on neutral or shared topics of interest.
2	For the last twelve months<br>2.1 As a minimum has freedom from conflict in family relationships (even if this includes the choice to minimise contact) **and**<br>2.2 Has mostly friendly interactions with those in the immediate millieu –<br>Is not prone to bullying, domination, exploitation or excessive isolation **and**.<br>2.3 Evidence of change/commitment/consistency to these patterns of relating is sustained over time – at least twelve months.<br>**Note: if** any of these are not achieved, **then** rate '3' or '4' as appropriate.
1	1.1 Is on good terms with all significant others, **or** else has found a safe way of getting on with them **and**<br>1.2 Is free of conflict with those in the immediate milieu (fellow patients / residents, formal and informal carers) **and** capable of friendship (mutual support) with some.<br>1.3 Where (**if**) dysfunction or conflict arises, (**then**) the person should be willing to seek help from the team in resolving this.<br>1.4 Where (**if**) there is an intimate relationship or pattern of relationships, (**then**) these are consensual **and** when dysfunction arises the person is/has been willing to seek help from the team in resolving this.
0	For a period of five years<br>0.1 has sustained good terms with all significant others, **or** else has found a safe way of getting on with them **and**.<br>0.2 Is free of conflict with those in the immediate milieu (fellow patients / residents, formal and informal carers) **and** is capable of friendship (mutual support) with some.<br>0.3 Where (**if**) dysfunction or conflict arises, (**then**) this is not part of a pattern of repetition.<br>0.4 Where (**if**) there is an intimate relationship or pattern of relationships, (**then**) these are consensual, **and** free of patterns of dysfunctional repetition.

Information Quality: 0=no information; 1=staff observation only; 2=interview and staff observation; 3=family informants; 4=medical or police records.

"successfully completed" means has attended at least 90% of scheduled sessions in a programme during which the patient engaged fully, has actively participated and has shown the ability to personalise the content by giving examples from own experience relating to him/herself.

"Engaged" means enters into and commits to, as shown by consistency and initiative, effort and supportiveness of the goals of an activity or programme.

© not to be copied or reproduced without permission.

DUNDRUM-4: RECOVERY ITEMS

This series of items is intended to provide a structured professional judgement instrument to assist the decision to move patients from higher to lower levels of therapeutic security. These items should always be used in conjunction with the previous series of items concerning the completion of treatment programmes DUNDRUM-3. These items should be seen as qualitatively different from the DUNDRUM-1 triage security and DUNDRUM-2 triage urgency items. The coding has a parallel however. As before, this is a structured professional judgement tool. It is not intended that these items should absolutely determine the appropriateness or timeliness of a move from higher to lower levels of security or a delay in transfer. These items are intended only as a guide to what is relevant to the decision making process. These items should be regarded as ‘dynamic’ and should be reassessed at intervals, perhaps every three months or six months. These items may function in an analogous way to the dynamic ‘protective’ scales in the START (Webster et al 2009) and SAPROF (de Vogel et al 2009).

As before there may be legal or administrative barriers to the movement of patients from one level of therapeutic security to a lower level, based on need. These judicial / administrative factors are not included as an item here because the items listed are intended to inform the decision making process, including advice given to those with legal or administrative control over such moves – variously according to jurisdiction these decision makers may be Government Ministers or their advisers, Mental Health Review Boards or simply the clinical directors of secure and community mental health services. Because judicial / administrative factors are not included, the Recovery items may be used as an audit tool for the appropriateness and timeliness of such movements.

Those who are mostly rated ‘4’ are unlikely to be ready for a move from a high secure to a medium secure setting, or to any setting at a lower level of security than their current one; those mostly rated ‘3’ should be ready for a move from a high secure to a medium secure setting; those mostly rated ‘2’ should be ready for a move from medium to low security; those mostly rated ‘1’ may be ready for a move to an open or community placement – though the availability of a high level of community support, structure and supervision, mandated if necessary by legally binding conditional discharge with a power of recall, may be a part of such a decision. Finally, those rated ‘0’ may be ready for an absolute legal discharge though this should be an individualised decision in all cases.

We note that in a recent study, the HCR-20 dynamic items, the ‘C’ and ‘R’ sub-scales correlated with the levels of security to which patients had been allocated (Muller-Isberner, Webster & Gretenkord 2007).

© not to be copied or reproduced without permission.

Recovery Item 1: Stability

The decision to move a person from high to medium security, or from medium to low (minimum) security, or from low to community or open placements, and eventually to recommend an absolute discharge may be critically influenced by the extent to which the person has been stable and predictable over time. Remission from symptoms (Frank et al 1991, Andreasen et all 2005) is not regarded here as essential for any step of progression from one level of care to the next lower level, though some improvement in mental state relevant to risk is implicit in the emphasis on 'relapse' and in particular the pattern of relapse. Similarly, more than symptoms of mental illness should be considered here. Problem behaviours such as violent or challenging behaviour, fire setting, stalking etc should also be considered from the point of view of desisting, stability and pattern of relapse.

‘Stability’ here is negated by evidence of relapse of positive symptoms, or evidence of violence or threatened violence to others rating above 4/6 on the DASA or requiring de-escalation, restraint, seclusion, additional medication or enhanced nursing observations.

Coding: R1. Stability

4	Has no stable or predictable pattern of<br>4.1 relapse of illness **or**<br>4.2 recurrence of problem behaviours.
3	For a period of one year -<br>3.1 Relapses **may be** abrupt over days **and** <u>unpredictable</u> **but**<br>3.2 has been stable for one year.
2	For a period of one year -<br>2.1 Relapses may be abrupt, over days, **but**<br>2.2 <u>are predictable</u> **and**<br>2.3 patient has been stable for one year. Age may be taken into account.
1	1.1 Relapses occur gradually over a period of weeks **and**<br>1.2 in response to known patterns or precipitants **and**<br>1.3 Signature signs and symptoms are known to carers **and**<br>1.4 acknowledged by patient. Age may be taken into account.
0	Over a period of five years:<br>0.1 no relapse or recurrence of problem behaviour **and**<br>0.2 relapse unlikely.<br>0.3 Advanced age may be taken into account

Information Quality: 0=no information; 1=staff observation only; 2=interview and staff observation; 3=family informants; 4=medical or police records.

© not to be copied or reproduced without permission.

Recovery Item 2: Insight

The most practical definition of insight is that given by Amador and David (1998) – dividing the concept into three independent elements – recognition of one's own illness, recognition that one's own symptoms such as delusions and hallucinations are the products of illness and acceptance of the benefits to one's self of medication and other aspects of treatment.

The emphasis here is on appreciation that imparted information is relevant to the person himself or herself (note how the MacArthur structured professional judgement tools for assessing functional mental capacity divide this into understanding, reasoning and appreciation Grisso & Appelbaum 1998). Adherence or compliance is also relevant as evidence for the practical reliability of this quality.

Aspects of openness and trust are rated elsewhere (R3 therapeutic rapport).

Coding: R2. Insight

4	4.1 Does not accept any aspect of own illness **and**<br>4.2 does not accept legal obligations **and**<br>4.3 does not engage actively in treatment or recovery oriented programmes.
3	Acknowledges own legal obligations as a minimum.
2	2.1 Accepts own legal obligations and accepts treatment **and**<br>2.2 is encouraged to do so by those friends or family who are most influential with him/her.
1	1.1 Realistic appraisal of own risk of relapse **and**<br>1.2 practical approach to relapse prevention **and**<br>1.3 family and friends, if involved, are aware and supportive **and**<br>1.4 has previously cooperated with relapse contingency plans when necessary.
0	Over a period of five years -<br>0.1 acknowledges own need for professional help and more general supports in maintaining recovery **and**.<br>0.2 cooperates with crisis contingency plans.<br>0.3 **If** in the event of relapse, **then** actively seeks help **and**<br>0.3 **If** previously relapsed **then** has cooperated with relapse contingency plans

Information Quality: 0=no information; 1=staff observation only; 2=interview and staff observation; 3=family informants; 4=medical or police records.

© not to be copied or reproduced without permission.

Recovery Item 3: Therapeutic Rapport

Working alliance and interpersonal trust are amongst the elements of therapeutic rapport. There is growing evidence that therapeutic rapport is one of the essential elements of meaningful outcome measurements for mental health. It has been suggested that all outcome measures in mental health (quality of life, needs, symptoms and satisfaction) assess a single tendency towards positive or negative appraisals (Hansen et al 2007). The same authors have described a correlation between attitudes to treatment and length of hospitalisation and between patient-rated unmet need and therapeutic alliance (Priebe 1995). · While this is commonly seen as a quality of the patient's attitude to the professional carers, it has a reciprocal which is best described as the trust the professional carers feel for the patient. The patient's sense of working alliance and interpersonal trust are aspects of an enduring disposition which non-the-less is amenable to change over the medium term.

Coding: R3. Therapeutic Rapport

4	4.1 Does not tolerate monitoring or supervision **or**<br>4.2 may seek to secrete, deceive or subvert **or**<br>4.3 Negative disposition towards carers and professionals generally.
3	3.1 Tolerates daily intrusions and constrictions of therapeutic security **and**<br>3.2 engages and participates in therapeutic and occupational programmes.
2	2.1 Capable of openness and trust with members of multi-disciplinary team **and**<br>2.2 capable of limited exploration of current mental state as related to risk.
1	1.1 Open and trusting with all members of multi-disciplinary team **and**<br>1.2 capable of communicating matters relevant to risk **and**<br>1.3 tolerates intrusion and restrictions on autonomy of treatment plan/ conditional discharge **and**<br>1.4 not excessively dependent on particular individuals i.e. is capable of transferring from one professional to another.
0	Over a period of five years:<br>0.1 maintains contact regularly and spontaneously **and**<br>0.2 is capable of transferring an open and communicative relationship from one professional to another at reasonable intervals.

Information Quality: 0=no information; 1=staff observation only; 2=interview and staff observation; 3=family informants; 4=medical or police records.

© not to be copied or reproduced without permission.

Recovery Item 4: Leave

The graded use of leave outside the secure perimeter is an important guide to the readiness for progression from one level of therapeutic security to the next. Leave is an essential part of the rehabilitation process and it is necessary to take ‘therapeutic risks’ to ensure that institutionalisation does not occur, or to remedy early signs of institutionalisation. Institutionalisation should not be confused with the negative or deficit state of schizophrenia, which is characterised by lack of motivation, poverty of thought and affective flattening. Institutionalisation is characterised by dependence on the routines of the hospital ward, loss of skills in the activities of daily living such as doing one’s own laundry, shopping and cooking for oneself and others, tending to one’s own living space and property, and knowledge of the outside world generally e.g. using modern coinage, public transport, dealing with official forms and offices. While this item is not a rating of institutionalisation or of negative symptoms, this item is included because the necessity of taking therapeutic risks when assessing suitability for leave is so central to the process of rehabilitation and recovery in a forensic setting.

© not to be copied or reproduced without permission.

Coding: R4. Leave

4	4.1 Represents such a high risk of absconding that can only leave a high secure setting under the close supervision of two or more members of staff **or**<br>4.2 Patient remains deluded or preoccupied with a former victim or category of victim and is still affectively motivated (e.g. angry, fearful) **or**<br>4.3 A known potential victim would be at risk of serious harm again if patient at liberty
3	3.1 Can safely visit a medium secure setting prior to moving there from a high secure setting **or**<br>3.2 can use occasional leave to visit hospitals, family or other private venues when accompanied by one member of staff.<br>3.3 can regularly use accompanied leave in the community with <u>two</u> members of staff<br>**except** when in relapse or when other indicators of risk are higher than usual
2	2.1 Can use accompanied leave in the grounds of the medium secure hospital most of the time **and**<br>2.2 can use accompanied leave in the community with <u>one</u> member of staff (if regularly requires two members of staff, rate '3').<br>**except** when in relapse or when other indicators of risk are higher than usual.
1	1.1 Has used unaccompanied leave in the community for at least six months.
0	For a period of at least five years<br>0.1 has lived in the community **and**<br>0.2 has tolerated home visits and / or visits to place of work by members of the mental health team, both planned and unannounced.

Information Quality: 0=no information; 1=staff observation only; 2=interview and staff observation; 3=family informants; 4=medical or police records.

© not to be copied or reproduced without permission.

Recovery Item 5: Dynamic Risk Items.

Modern structured professional judgment instruments such as the HCR-20/HCR-V3 ‘Clinical’ or current items and the HCR-20/HCR-V3 ‘Risk’ or future items are combined as ‘dynamic’ indicators of change over time (Webster et al 1997). The S-RAMM current and future items (Bouch & Marshall 2003), START (Webster et al 2009) and SAPROF (de Vogel et al 2009) may also describe these risk factors which are amenable to change. The HCR-20/HCR-V3 ‘Risk’ or future items are usually rated for the eventuality of remaining in their present placement (‘in’) or moving to a less secure or open / community placement (‘out’). In general, if there is an obvious difference in the ratings for ‘in’ and ‘out’ then a move to a less secure place would increase the risk of violence.

As for Item T7, the rating for this item is not based on artificial actuarially calculated scores and probabilities. Instead the ratings are based on profiles of change over time.

© not to be copied or reproduced without permission.

Coding: R5. Dynamic Risk Items

4	4.1 There is a score of ‘8’ or more on ‘R’ items for a move from present level of security to the proposed next lowest level, **or**<br>4.2 There is a substantial difference (4 or more) between the ‘in’ and ‘out’ scores for ‘risk/future’ items (HCR-20 & HCR-V3 R1 to R5), when computed for any move to a lower level of security than the current placement **or**.<br>4.3 HCR-20 C2 negative attitudes/ HCR-V3 violent ideation or intent is rated positive ('2').
3	3.1 There is a score of ‘8’ or more on ‘C’ items **or**<br>3.2 **if** rated '2' on HCR-20 C2 negative attitudes / HCR-V3 violent ideation, **then** rate 4.
2	2.1 The move from medium to low therapeutic security may increase exposure to destabilisers (HCR-20 & HCR-V3 R2) and certain types of stress (HCR-20 & HCR-V3 R5), if so this should inhibit such a move while these issues are dealt with either through further psychological treatment, through addressing the choice of setting or level of support to be provided on moving.
1	1.1 The move from low secure to open or community places may increase exposure to destabilisers **and**<br>1.2 The dynamic scores should be equally low ‘in’ and ‘out’, while negative attitudes (HCR-20 C2) or violent ideation or intent (HCR-V3 H9 & C2) and impulsivity (HCR-20 C4) or instability (HCR-V3 C4) particularly would inhibit such a move **and**<br>1.3 Active symptoms (HCR-20 C3/HCR-V3 C3), **if** they remain should be much reduced and stabilised. See R3 ‘Rapport’ regarding insight (HCR-20 & HCR-V3 C1). Plans lack feasibility (HCR-20 R1) should be regarded as particularly important.
0	For a period of five years<br>0.1 If the dynamic items have remained low and stable **and**<br>0.2 **if** the Current / present items are similarly stable and low, **then** the transition from conditional discharge in the community to absolute discharge may be considered.<br>0.3 It **may be** that this can only safely be accomplished where there is consistent evidence of remission of symptoms (e.g. HCR-20/HCR-V3 C3=0 or Andreasen criteria for remission).

Information Quality: 0=no information; 1=staff observation only; 2=interview and staff observation; 3=family informants; 4=medical or police records.

© not to be copied or reproduced without permission.

Recovery Item 6: Victim Sensitivity Items.

This item presents special problems in balancing the rights and expectations of victims and patients. As a minimum, there should be a requirement that no fear or distress is afforded to the reasonable former victim or surviving relative of the victim. Some communities may be welcoming to the return of the patient, but some may not. If this were to engender a media campaign it would not be in the interests of the patient. An unsuccessful return to the former home community would have serious consequences for the future recovery of the patient. Accordingly, an essential part of the recovery process is the extent to which the needs of victims or their surviving relatives can be assessed and accommodated. This may be done by members of one of the other multi-disciplinary teams and/or a specialist victim support service making contact and offering information, support and advice, while avoiding breeching confidentiality. The needs of the victims can be incorporated into treatment and management plans, and conditions for leave and discharge. A continuing preoccupation with the former victim or with a predictable category of victim should also be rated here.

© not to be copied or reproduced without permission.

Coding: R6. Victim Sensitivity Items

4	4.1 Victim or survivors remain actively engaged in petitioning against the movement of the patient or increase in access to the community **or**<br>4.2 Media interest remains active, stigmatising and would pose a risk to the patient **or**<br>4.3 Preoccupation with victim or category of victims remains pervasive.
3	3.1 Patient's preoccupation with specific victim or category of victims is encapsulated and no longer pervasive **and**<br>3.2 Victim or survivors are engaged in a process of liaison which respects confidentiality and the needs of both victim and patient, **and**<br>3.3 Victim or survivors would be upset / traumatised by contact but lesser harm than the original offence even if patient was in community **and**<br>3.4 Media interest is no longer active or intrusive but would still be hostile.
2	2.1 Patient is capable of recognising the potential for hurt to the victim or category of victims. If at liberty would not represent a threat to them **and**<br>2.2 Victim or survivors can be accommodated by reasonable conditions and restrictions on the movements of the patient outside the hospital e.g. exclusion zones **and**<br>2.3 Victim would not be at risk of harm if patient was at liberty **or**<br>2.4 Media interest is no longer likely.
1	1.1 Patient accepts and complies with conditions regarding non-contact with victim or surviving relatives of victim or category of victims as appropriate **and**<br>1.2 Victim or survivors can be accommodated by reasonable conditions and restrictions on the movements of the patient and these have been observed by the patient while on leave from the hospital **and**<br>1.3 Victim or survivors would not be upset by patient being in community, includes geographic exclusions to prevent accidental meeting **and**<br>1.4 Media interest is no longer likely and patient should be able to live anonymously in the proposed community location for discharge.
0	For a period of five years<br>0.1 Patient is capable of remorse for harm done to the victim and victim's relatives **and**<br>0.2 Victim or survivors have not been actively involved or are reconciled (e.g. intra-family victims) **and**<br>0.3 Media interest has not been active for five years and patient has been living anonymously in the community

Information Quality: 0=no information; 1=staff observation only; 2=interview and staff observation; 3=family informants; 4=medical or police records.

© not to be copied or reproduced without permission.

DUNDRUM TOOLKIT

SELF RATED VERSION

© not to be copied or reproduced without permission.

How to use this toolkit

The DUNDRUM Quartet is a new measure of your progress. Part of this assessment will be completed by your team. However it is very important to us that we ask you for your own opinions. We do not feel your needs assessment would be complete without your own input. This is why we are asking that each service user would fill out this form.

The first part of this assessment is called the “Programme Completion” section. This section asks questions about the treatment programmes you may have taken part in or completed. Examples might include WRAP (Wellness Recovery Action Plan) or Wellness or the Drugs and Alcohol programmes.

The second part of this assessment is called the “Recovery” section. This section looks at how you think you are recovering, for example it asks what your view of your own health is, what leave you think you should have and how you get on with your team.

How to complete this form:

You simply rate yourself 0,1,2,3 or 4, for each item, depending on which description you feel is most appropriate to you at this time.

Don’t worry if you think your team may score differently to how you have scored yourself, it is your opinion we are asking for.

If you have any comments or questions about this risk assessment, we would be happy to hear them.

Name:__________________________________

Unit:____________________________________

Date:____________________________________

© not to be copied or reproduced without permission.

Self-rate: P1. Physical Health

Please tick ✓ which box either 0, 1, 2, 3, 4 which applies most to you

**4**	I have not yet successfully completed* any programme concerning physical health awareness.<br>I do not wish to change my lifestyle.<br>I do not want to participate in physical health activities<br>Staff provide for my basic activities of daily living and self care.	
**3**	I understand the need to take care of my physical health<br>I participate in healthy lifestyles programmes or activities when prompted<br>I have attended GP when prompted to do so for regular check ups.	
**2**	I have participated in education programmes regarding physical health (e.g. 'Solutions for Wellness' or similar programme).<br>I am interested cooking healthy food<br>Take regular exercise.<br>Sometimes I need prompting to keep me motivated	
**1**	I manage my own medication<br>I take an active interest in my physical health<br>I participate in regular exercise and healthy eating.<br>Occasionally I need prompting to follow a healthy lifestyle	
**0**	For a period of five years I have taken responsibility for my own active recovery and personal physical health,<br>I regularly attend my family doctor,<br>I manage my own diet and my own medication and have a regular pattern of self-care.<br>I participate in regular physical exercise	

*** "Successfully completed" means has attended at least 90% of scheduled sessions in a programme during which you engaged fully, actively participated and showed the ability to personalise the content by giving examples from your own experience relating to yourself.**

*** "Engaged" means you entered into the sessions of the programme and committed to making them work for you, as shown by your consistency and initiative, effort and supportiveness of the goals of the activity or programme.**

© not to be copied or reproduced without permission.

© not to be copied or reproduced without permission.

Self-rate: P2: Mental Health:

This item rates your progress in actively managing your mental health. A first step for most people would be an education programme regarding physical and mental health. This would be followed by a programme specifically focusing on mental health and recovery, for example WRAP.

Please tick ✓ which box either 0, 1, 2, 3, 4 that applies most to you

4	I don't think I have a mental illness<br>I take medication because I'm in hospital.<br>I do not wish to take part in information programmes.	
3	I have participated in information programmes about mental health and I have completed a 'wellness programme" or equivalent.<br>I am interested in learning more about my mental health and how to prevent me getting ill again. I take medication that is prescribed but not sure if I want to continue with medication	
2	I have remained well for the past twelve months and<br>I am aware of the need to maintain a balance in my life.(friends, family, occupation and leisure)<br>I occasionally need reminding from nursing staff to take my medication and attend my appointments	
1	I feel confident that I manage my own mental health well<br>I have a network of friends and family as well as support from mental health professionals.<br>I do not need reminding to take my medication and attend my appointments<br>I have a regular pattern and routine of activities over the day, week and year. I know and recognise my early warning signs of relapse<br>I actively seek out support<br>I have developed my own WRAP programme.	
0	My mental health has remained stable for the past five years.<br>I have a good knowledge of my mental health needs	

© not to be copied or reproduced without permission.

<u>Self-rate:</u> P3. Drugs and Alcohol

Many people struggle with substance misuse problems alongside their mental illness. Research shows us that having a severe mental illness together with using drugs and alcohol leaves us at greater risk of being violent. This is why abstinence is promoted in forensic mental health services. Some people may believe that a return to 'controlled drinking' after a period of dependence is acceptable. However there is not much evidence to support this and would not be recommended to assist you in your recovery from mental illness.

Please tick ✓ which box either 0, 1, 2, 3, 4 that applies most to you

4	I do not wish to participate in an information programme about drugs and /or alcohol<br>I do not wish to stop taking drugs or drinking alcohol	
3	I have completed an education programme regarding drugs and alcohol.<br>I'm unsure if I want to give up drugs<br>I'm unsure if I want to give up alcohol.<br>I have not used illegal drugs while in hospital	
2	I have successfully completed a full drug and alcohol recovery programme.<br>I am working towards abstinence (action) e.g. by limiting/ending contact with former circle of users.<br>I have been abstinent from drugs and/or alcohol for at least twelve months e.g sustained abstinence.<br>I attend self groups<br>I still need continued prompting / guidance.	
1	I have been fully engaged with follow-up / maintenance programmes as appropriate.<br>No positive drug screens.<br>I cope with 'slips' by seeking help.<br>I have regular patterns and routines that support me avoid risk situations<br>I get self-confidence from my identity as an abstinent person or as a person in recovery.<br>I attend self help groups	
0	I have never had a drug or alcohol problem<br>For a period of five years I have been totally abstinent<br>I recognise situations that may lead to relapse	

© not to be copied or reproduced without permission.

<u>Self-rate: </u>P4: Problem Behaviours:

Problem behaviours are behaviours that may cause you to put yourself or other people at risk of harm. Examples of courses or programmes that address problem behaviours would include ETS (Enhanced thinking skills), CALM or DBT (Dialectical behaviour therapy), and '5WH' programmes (who, what, why, when, where) done with the Book of Evidence.

Please tick ✓ which box either 0, 1, 2, 3, 4 that applies most to you

4	I do not wish to address any issues in relation to my behaviour<br>I do not wish to change the way I behave	
3	I have participated in programmes in relation to developing a greater understanding of my high risk behaviours (meta-cognitive training, enhanced thinking skills or 'balance' programme (DBT modules).<br>I understand that there are some behaviour I have that need to change in order to manage situations that could lead to problem behaviour.	
2	I have successfully completed programmes to address problem behaviours e.g. anger management, healthy relationships and healthy sexual functioning,<br>I have an understanding of my previous problem behaviours.<br>Over the past twelve months there has been no episode of problem behaviour that required staff intervention	
1	I cope with behavioural 'slips' or new stresses by seeking appropriate help from the team in a timely way.<br>I have gained confidence increased self esteem s associated with avoiding problem behaviours.	
0	For the past five years I have not been involved in any behaviours that poses a risk to myself and others<br>I have an awareness of situations that may lead to violent or aggressive behaviour<br>I have participated in programmes that helps me understand my previous problem behaviours<br>I have a network of support to discuss stressful situations	

© not to be copied or reproduced without permission.

Self-rate: P5. Self-Care and Activities of Daily Living

This refers to courses you may complete to help you develop skills to increase your independence e.g. self-catering, including budgeting skills, shopping and use of public transport.

Please tick ✓ which box either 0, 1, 2, 3, 4 that applies most to you

4	I have not yet completed any programme concerning self-care or basic social skills, activities of daily living<br>I do not wish to participate in groups<br>Staff can provide for my needs	
3	I have participated in assessments in relation to daily living tasks<br>I am interested in improving my skills<br>I find it difficult to follow through with tasks	
2	I have successfully completed OT courses on self-catering, budgeting, shopping and the use of public transport.<br>My skills have improved over the last twelve months<br>I am able to work safely in the kitchen or workshop<br>I occasionally need prompting to complete tasks	
1	I am engaged with a well-balanced daily and weekly programme of self-care, occupation and leisure.<br>I feel a sense of pride and increased self-confidence<br>I feel a sense of dignity and respect	
0	I have maintained an interest in active recovery and personal mental and physical health, for a period of five years<br>I have a regular pattern of self-care, occupation and leisure.<br>I have a network of informal as well as professional supports and carers.	

© not to be copied or reproduced without permission.

<u>Self-rate: </u>P6: Education, Occupation and Creativity

This item asks about your education and interests that you engage in. Examples may include literacy courses, walking groups and film clubs.

Please tick ✓ which box either 0, 1, 2, 3, 4 that applies most to you

4	I do not enjoy courses<br>I have no interest in activities or exploring occupations. I attend activities and programmes because I have to.	
3	I have attended some short courses<br>I sometimes find it difficult to stay motivated<br>I need encouragement and support to try new things, as I am not really sure of I am good at doing anything.	
2	I have a timetable that provides a structure to my day<br>Over the past 12 months I have participated in a range of educational and leisure programmes<br>I take an active interest in current affairs	
1	I have regular a timetable of varied leisure activities<br>I participate in educational courses or paid un paid work.<br>I enjoy keeping busy	
0	For a period of five years I have maintained a regular pattern of self-care, occupation and leisure.<br>I have a wide a range of interests and activities including education, work (paid or un-paid), sport, creativity<br>I am interested in current affairs	

© not to be copied or reproduced without permission.

<u>Self-rate:</u> P7 Family and Social Networks, Friendship and Intimacy

This item asks your opinion on how you relate to those around you, both friends and family members.

Please tick ✓ which box either 0, 1, 2, 3, 4 that applies most to you

4	I rarely talk to my peers<br>I prefer to be left alone<br>Other patients and some staff 'wind me up'<br>I do not wish to see family or friends	
3	I tend not to engage much with my peers (live and let live)<br>I engage with staff when they approach me.<br>I tend to isolate myself from others	
2	My family relationships are free from conflict<br>I generally get on well with neighbours, residents or informal carers<br>For the past twelve months I have not been involved in any negative behaviour with my peers (Bullying domination, exploitation)<br>I interact well with people around me	
1	I am on good terms with my family and all significant others.<br>I have developed friendships that are mutually supportive.<br>If conflict arises I am able to recognise it and seek help in resolving it appropriately.<br>My intimate relationships are respectful and consensual.	
0	For a period of five years I have sustained good terms with all significant others, or else I have found a safe way of getting on with them.<br>I have no ongoing conflict with neighbours, residents or informal carers and I am capable of friendship (mutual support) with some.<br>Where conflict with others arises I am able to manage it successfully.<br>My intimate relationships are respectful and consensual.	

© not to be copied or reproduced without permission.

<u>Self-rate:</u> Recovery Item 1: Stability

This refers to how long you have been well without a relapse of symptoms e.g. hallucinations, experiencing depressive or manic episodes, or evidence of violence or threatened violence to others requiring de-escalation, restraint, seclusion, additional medication or enhanced nursing observations.

Please tick ✓ which box either 0, 1, 2, 3, 4 that applies most to you

4	I don't know when I am relapsing	
3	I have not had a relapse of illness for 12 months<br>I am not aware of any particular signs or symptoms prior to relapse<br>My family and those close to me are not aware of any particular signs or symptoms prior to my relapses OR<br>my family have not been able to get help for me when I was relapsing	
2	My relapses occur quickly over days but are predictable<br>I am aware of my signature symptoms of relapse<br>My family and those close to me are aware of my relapse pattern	
1	My relapses in the past has always been gradual and happen slowly	
0	I have not experienced any relapse of symptoms over the last five years.<br>Over the last five years in the community I have always remained engaged with my care and treatment plan.	

© not to be copied or reproduced without permission.

<u>Self-rate:</u> Recovery Item 2: Insight

This item asks your opinion on whether or not you believe that you have an illness or need treatment.

Please tick ✓ which box either 0, 1, 2, 3, 4 that applies most to you

4	I do not think I should be in hospital<br>I do not believe that I have a mental illness<br>I do not wish to become involved in any treatment programme for mental health	
3	I believe I do not need treatment<br>I will take medication because the law obliges me to.	
2	I go to therapeutic groups and some of what I learn there applies to me, but some does not.	
1	I think that I need medication and I have learned useful things in therapeutic groups<br>My family/friends are aware of and supportive of my treatment plan<br>My relapse prevention plan has worked well in the past (where relevant)	
0	Over the past five years:<br>I am aware of my crisis plan in the event of relapse or difficulties.<br>I have always asked for help from my treating team or family and friends if I am relapsing.	

© not to be copied or reproduced without permission.

<u>Self-rate:</u> Recovery Item 3: Therapeutic Rapport

This item asks about whether you trust your treating team and whether or not you think they act in your best interests.

Please tick ✓ which box either 0, 1, 2, 3, 4 that applies most to you

4	I find it difficult to discuss my concerns with members of the team.<br>I prefer when staff leave me alone<br>I feel most of the staff are against me.<br>Answering questions irritates me	
3	I do what my team asks me to do.<br>I find it difficult to be open with some members of my team<br>I do not trust all of the staff	
2	Sometimes I worry about raising issues with my team in, case it slows down my progress	
1	I find it easy to discuss issues with most members of mental health team<br>I am able to discuss issues relevant to my care plan<br>I actively participate in developing my care plan.<br>I believe my team respects me	
0	For the past five years<br>I have maintained a good relationship with my mental health team<br>I have trust in my mental health team members<br>I cope well with changes in the team<br>I am happy for members of the team to visit me at home.	

© not to be copied or reproduced without permission.

<u>Self-rate:</u> Recovery Item 4: Leave

Leave is an essential part of your rehabilitation. Leave helps to prevent people becoming dependent on the hospital and losing their skills.

Please tick ✓ which box either 0, 1, 2, 3, 4 that applies most to you

4	I believe that I am ready to have unaccompanied leave from the hospital. My team do not think that I am ready for any leave from the hospital.	
3	I believe that I am ready to have regular leave from the hospital. My team think that I am able to have occasional visits from the hospital to attend special occasions or hospital appointments accompanied by staff.	
2	I am ready for regular accompanied leave in the hospital grounds and the community<br>It would be helpful to have a member of staff with me	
1	I have been getting unaccompanied leave from the hospital, in the community, for at least six months without incident.	
0	For at least the past five years I have been able to live independently in the community without any setbacks	

© not to be copied or reproduced without permission.

<u>Self-rate:</u> Recovery Item 5: Dynamic Risk Items.
This item asks your opinion on the risk assessments e.g. the HCR-20 and SRAMM that your team have completed for you.

Please tick ✓ which box either 0, 1, 2, 3, 4 that applies most to you

Coding: R5. Dynamic Risk Items

4	I do not think that I pose a risk to either to myself or other people.<br>I am not aware why I am in this forensic hospital as opposed to a general community hospital.	.
3	My team think I am a high risk of harm to others but I don't think I am as high a risk as they say.<br>I participate in my risk assessments	
2	I participate in my risk assessment.<br>I need to complete additional therapeutic groups in order to better manage my risk	
1	I participate in my risk assessment.<br>My current identified risks of violence to other people are low and decreasing over the past year	
0	As part of my care plan I participate in my risk assessment<br>and my risk has remained low for the past 5 years	

© not to be copied or reproduced without permission.

Self-rate: Recovery Item 6: Public protection items.
This item refers to balancing the rights and expectations of victims with your rights and expectations.

Please tick ✓ which box either 0, 1, 2, 3, 4 that applies most to you

Coding: R6. Victim sensitivity items

4	There are people outside that I have a score to settle things with, or I would lose my temper if I met them. The people I had problems with in the past should be frightened if I met them. I would have to defend myself against them.<br>There are people outside who are against my leaving the hospital. Their views should not be taken into account.<br>In the last year the media are still publishing stories about me.	
3	I sometimes think about the people who used to bother me, when I think about them I am not as upset.<br>There are people outside who may be against my leaving the hospital. Their views could be taken into account.<br>I think the media might still be interested in stories about me.	
2	If I met the people I've had problems with in the past I wouldn't want to do anything to hurt or upset them.<br>There are people outside who may be upset about my leaving the hospital. Their views should be taken into account<br>I don't think the media would still be interested in stories about me.	
1	I would try to ensure that I kept away from people who would be upset to see me.<br>There are people outside who may be upset about my leaving the hospital. Their views have been taken into account<br>I am confident that the newspapers wouldn't write about me if I was in the community.	
0	For the past five years while accessing the community I have avoided contact with people who might be upset to see me.<br>For five years there have been no signs that any people outside might be upset about my being in the community.<br>In the last five years there has been no media interest in my story while I have been living in the community.	

© not to be copied or reproduced without permission.

REFERENCES

Abidin Z, Davoren M, Naughton L, Gibbons O, Nulty A, Kennedy HG. Susceptibility (risk and protective) factors for in-patient violence and self-harm: prospective study of structured professional judgement instruments START and SAPROF, DUNDRUM-3 and DUNDRUM-4 in forensic mental health services. *BMC Psychiatry* 2013, **13**:197 doi:10.1186/1471-244X-13-197

American Psychiatric Association. Diagnostic and Statistical Manual of Mental disorders 4th edition. APA: Washington DC, 1994.

Amador XF, David AS (eds). Insight and Psychosis. Oxford, Oxford University Press 1998.

Andreasen NC, Carpenter WT, Kane JM, Lasser RA, Marder SR, Weinberger DR. Remission in schizophrenia: proposed criteria and rationale for consensus. Americal Journal of Psychiatry 2005. 162:441-449.

Andresen, R., Oades, L., & Caputi, P. (2003). The experience of recovery from schizophrenia: towards an empirically-validated stage model. *Australian and New Zealand Journal of Psychiatry,* 37, 586–594 [see also http://www.uow.edu.au/health/iimh/index.html]

Andrews DA & Bonta J (1995) LSI-R: The Level of Service Inventory-Revised. Toronto, Mental Health systems.

Appleby L, Shaw J, Sherratt J et al. (2001) Safety First: Five-year report of the National Confidential Inquiry into Suicide and Homicide by people with mental illness. London: Department of Health.

Beautrais AL (2001) Suicides and serous suicide attempts: two populations or one? Psychological Medicine 31: 837-845.

Berecochea JE & Gibbs JL (1991) Inmate classification: a correctional programme that works? Evaluation Review 15, 333-363.

Bouch J & Marshall JJ (2003) S-RAMM: Suicide Risk Assessment and Management Manual (Research Edition) Cognitive Centre Foundation, Vale of Glamorgan.

Brown CSH & Lloyd K. (2002) Comparing clinical risk assessments using operationalised criteria. Acta Psychiatrica Scand 2002 supplement 142, 148-151

Brown CSH & Lloyd K (2008) OPRISK: a structured checklist assessing security needs for mentally disordered offenders referred to high security hospital. Criminal Behaviour & Mental Health 18, 190-202

© not to be copied or reproduced without permission.

Cohen A, Eastman N. Assessing Forensic Mental Health Need: Policy, Theory and Research. London: Gaskell Press, 2000.

Coid J, Kahtan N. 2000. An instrument to measure the security needs of patients in medium security. *Journal of Forensic Psychiatry* **11**, 119-134

H Collins & R Evans *Rethinking Expertise,* Chicago, University of Chicago Press 2007.

Collins M, Davies S. The Security Needs Assessment Profile: a multi-dimensional approach to measuring security needs. International Journal of Forensic Mental Health 2005 4(1) 39-62.

M Davoren**,** Z Abidin**,** L Naughton**,** O Gibbons**,** A Nulty**,** B Wright**.** HG Kennedy. Prospective study of factors influencing conditional discharge from a forensic hospital: the DUNDRUM-3 programme completion and DUNDRUM-4 recovery structured professional judgement instruments and risk. *BMC Psychiatry* 2013a, **13**:185 doi:10.1186/1471-244X-13-185

Davoren M**,** Abidin Z**,** Naughton L**,** Gibbons O**,** Nulty A**,** Wright B**.** Kennedy HG. Prospective study of factors influencing conditional discharge from a forensic hospital: the DUNDRUM-3 programme completion and DUNDRUM-4 recovery structured professional judgement instruments and risk. *BMC Psychiatry* 2013b, **13**:185 doi:10.1186/1471-244X-13-185

Dernevik M, Grann M, Johansson S. Violent behaviour in forensic psychiatry patients: Risk assessment and different risk-management levels using the HCR-20. Psychology, Crime & Law 2002; 8(1): 93-102;

de Vogel V, de Ruiter C, Bouman Y, de Vries Robbe M. SAPROF: Structured Assessment of PROtective Factors for violence risk. Guidelines fo the assessment of protective factors for violence risk. Rnglish Version. 2009. Utrecht. Forum Educatief.

DiClemente CC, Prochaska JO, Fairhurst SK, Velicer WF, Velasquez MM, Rossi JR. The process of smoking cessation: an analysis of pre-contemplation, contemplation and preparation stages of change. Journal of Consulting and Clinical Psychology 1991. 59: 295-304.

Douglas KS, Webster CD, Hart SD, Eaves D, Ogloff JRP. HCR-20- violence risk management companion guide. Burnaby, British Columbia: Mental Health, Law and Policy Institute, Simon Fraser University, 2001

Eastman N. & Bellamy S. .*Admission Criteria for Secure Services Schedule (ACSeSS).* St Georges Hospital Medical School 1998

© not to be copied or reproduced without permission.

Fagan J, Papaconstantinou A, Ijaz A, Lynch A, O'Neill H, Kennedy HG. The Suicide Risk Assessment and Management Manual (S-RAMM) Validation Study II: Prospective Study of a Structured Professional Judgement Tool for Suicide Risk Assessment. *Irish Journal of Psychological Medicine* 2009, 26(3), *107-113*

Flynn G, O'Neill C, McInerney C, Kennedy HG. The DUNDRUM-1 structured professional judgment for triage to appropriate levels of therapeutic security: retrospective-cohort validation study**.** *BMC Psychiatry* 2011a, **11**:43

Flynn G, O'Neill C, Kennedy HG. DUNDRUM-2: Prospective validation of a structured professional judgment instrument assessing priority for admission from the waiting list for a Forensic Mental Health Hospital. *BMC Research Notes* 2011b, **4**:230

Frank E, Prien R, Jarrett R, Keller M, Kupfer D, Lavori P, et al. Conceptualisation and rationale for consensus definitions of terms in major depressive disorder: Remission, recovery, relapse and recurrence. Arch Gen Psychiatry. 1991;48:851-5.

Grisso T., & Appelbaum P.S. (1998): *Assessing Competence to Consent to Treatment.* Oxford: Oxford University Press.

Grubin D, Kelly P, Brunsden C (2001) Linking serious sexual assaults through behaviour. Home Office Research Study 215. Home Office Research, Development and Statistics Directorate, London. http://www.homeoffice.gov.uk/rds/pdfs/hors215.pdf

Hansson, L; Bjorkman, T; Priebe, S. Are important patient-rated outcomes in community mental health care explained by only one factor? Acta Psychiatrica Scandinavica. 2007, 116(2), 113-118.

Ijaz A, Papaconstantinou A, O'Neill H, Kennedy HG. The Suicide Risk Assessment and Management Manual (S-RAMM) Validation Study I: Inter-Rater Reliability, Internal Consistency, and Discriminatory Capacity. Irish Journal of Psychological Medicine 2009; 26(2):54-58.

Jadad AR, O'Grady L. (2008). How should health be defined? British Medical Journal 337, a2900. doi:10.1136/bmj.a2900.

James DV, Kerrigan TR, Forfar R, Farnham FR, Preston LF. The Fixated Threat Assessment Centre: preventing harm and facilitating care. Journal of Forensic Psychiatry and Psychology 2010. 1-16. doi 10.1080/14789941003596982.

Kay SR, Fiszbein A, Opler LA. The positive and negative syndrome scale (PANSS) for schizophrenia. Schiz Bull 1987; 13: 261-277.

Kennedy HG. Therapeutic Uses of Security: mapping forensic mental health services by stratifying risk. . Advances in Psychiatric Treatment 2002. 8: 433-443

© not to be copied or reproduced without permission.

Langan PA, Levin DJ. Recidivism of prisoners released in 1994. Bureau of Justice Special Report, June 2002 NCJ 193427. Washington D.C. Bureau of Justice Statistics.

A.H. Maslow, A Theory of Human Motivation, Psychological Review 1943. 50(4):370-96

Mullen PE, Pathe M, Purcell R. Stalkers and their victims (2nd edition). New York: Cambridge University Press 2009.

Muller-Isberner M, Webster CD, Gretenkord L. Measuring progress in hospital order treatment: relationship between levels of security and C and R scores of the HCR-20. Int J Forensic Men Health 2007; 6: 113-121

O'Dwyer S, Davoren M, Abidin Z, Doyle E, McDonnell K, Kennedy HG. The DUNDRUM Quartet: validation of structured professional judgement instruments DUNDRUM-3 assessment of programme completion and DUNDRUM-4 assessment of recovery in forensic mental health services. *BMC Research Notes* 2011, **4**:229

O'Neill C, Heffernan O, Goggins R, Corcoran C, Linehan S, Duffy D, O'Neill H, Smith C, Kennedy HG. Long-stay forensic psychiatric inpatients in the republic of Ireland: aggregated needs assessment. Irish Journal of Psychological Medicine 2003, 20: 119-125

Pierzchniak P, Farnham F, deTaranto N D Bull, H Gill, P Bester, A McCallum, Kennedy H. Assessing the needs of patients in secure settings: a multidisciplinary approach. *Journal of Forensic Psychiatry* 1999;**10**(2)**:**343-354

.Priebe, S; Gruyters, T. Patients' assessment of treatment predicting outcome. Schizophrenia Bulletin. Vol 21(1) 1995, 87-94

Prochaska JO, DiClemente CC. Stages and processes of self-change of smoking: towards an integrative model of change. Journal of Consulting and Clinical Psychology 1983, 51:350-395.

Pillay SM, Oliver B, Butler L, Kennedy HG. Risk stratification and the care pathway Irish Journal of Psychological Medicine 2008; 25(4): 123-127

Reed V, Woods P, Robinson D. The Behavioural Status Index (BSI): A Life Skills Assessment for Selecting and Monitoring Therapy in Mental Health Care. Psychometric Press 2000.

Rice ME, Harris GT (1996) Predicting the recidivism of mentally disordered fire setters. Journal of Interpersonal Violence 11, 364-375.

Scott PD (1977) Assessing dangerousness in criminals. British Journal of Psychiatry 131, 127-142

© not to be copied or reproduced without permission.

Shaw J, Davies J, Morey H (2001) An assessment of the security, dependency and treatment needs of all patients in secure services in a UK health region. Journal of Forensic Psychiatry 12, 610-637.

Shaw SH (1973) The Dangerousness of dangerousness. Medicine Science and the Law 13, 120-126

Stander J, Farrington DP, Hill G, Altham PME (1989) Markov chain analysis and specialization in criminal careers. British Journal of Criminology 29, 317-335.

Sugarman PA, Walker L (2004) HoNOS-SECURE version 2. London: Royal College of Psychiatrists College Research and Teaching Unit

Thomas S, Harty MA, Parrott J, McCrone P, Slade M, Thornicroft G. (2003). CANFOR: Camberwell Assessment of Need – Forensic Version. London: Gaskell.

Tracy PE, Wolfgang ME, Figlio RM (1990) Delinquency careers in two birth cohorts. New York, Plenum.

Walker N McCabe S (1973). Crime and Insanity in England: Two – New Solutions and New Problems. Edinburgh, Edinburgh University Press.

Webster CS, Douglas KS, Eaves D, Hart SD. (1997) HCR-20: Assessing risk for violence, version 2. Burnaby, British Columbia: Simon Fraser University.

Webster CD, Martin M-L, Brink J, Nicholls TL, Desmarais SL. Short-Term Assessment of Risk and Treatability (START). Version 1.1. 2009. Coquitlam BC. Forensic Psychiatry Services Commission & Hamilton. St Joseph's Healthcare. http://www.bcmhas.ca/Research/Research_START.htm

Weeks G, Slade M, Hayward M. A UK validation of the stages of recovery instrument. International Journal of Social Psychiatry 2010, doi:10.1177/0020764010365414

World Health Organisation (1946) Definition of Health, In: Preamble to the Constitution of the World Health Organization as adopted by the International Health Conference, New York, 19-22 June, 1946; signed on 22 July 1946 by the representatives of 61 States (Official Records of the World Health Organization, no. 2, p. 100) and entered into force on 7 April 1948

World Health Organisation (1986) Ottawa Charter for Health Promotion First International Conference on Health Promotion Ottawa, 21 November 1986 - WHO/HPR/HEP/95.1 http://www.who.int/hpr/NPH/docs/ottawa_charter_hp.pdf

Wykes T, Sturt E. The measurement of social behaviour in psychiatric patients: an assessment of the reliability and validity of the SBS schedule. *British Journal of Psychiatry* 1986;**148:**1-11

© not to be copied or reproduced without permission.

APPENDIX A: Preadmission Triage Instrument

Patient Name		
Rater name/s		
Date of assessment		
Patient Location (Tick)		***Community Forensic Patient***
		Remand Prisoner
		Sentenced Prisoner
		Forensic patient: Proposed higher level of security
		Forensic patient: Proposed lower level of security

Dundrum Toolkit

Instructions:

- **All ratings are based on accompanying manual.**
- **Triage security and Urgency items to be completed prior to placing individual on waiting list.**
- **Urgency items to be revised on a weekly basis for Monday triage meeting until admitted or taken off waiting list.**

© not to be copied or reproduced without permission.

Note: The DUNDRUM-1 11-item score may be used as an audit tool or for benchmarking. For many purposes, a score consisting of nine items excluding the two self-harm items, if divided by 9, gives a score that can be used to describe patient cohorts in terms of mean need for therapeutic security, where a mean greater than 3 indicates high security, a mean greater than 2 indicates medium security, a mean greater than 1 indicates low security (psychiatric intensive care or longer term low security), and a mean between 0 and 1 indicates open hospital or community supported placement.

		SCORE				
DUNDRUM-1:TRIAGE SECURITY ITEMS		***0***	***1***	***2***	***3***	***4***
S1	*Seriousness of violence*					
S2	*Seriousness of self-harm*					
S3	*Immediacy of risk of violence*					
S4	*Immediacy of risk of suicide/ self harm*					
S5	*Specialist forensic need*					
S6	*Absconding / eloping*					
S7	*Preventing access*					
S8	*Victim sensitivity/public confidence issues*					
S9	*Complex Risk of Violence*					
S10	*Institutional behaviour*					
S11	*Legal process*					
	Subtotal 11 items TS1-TS9					
	Subtotal 9 items omit TS2 & TS4					
	DUNDRUM-2: TRIAGE URGENCY ITEMS	***0***	***1***	***2***	***3***	***4***
U 1	*Current Location*					
U2	*Mental Health*					
U3	*Suicide Prevention*					
U4	*Humanitarian*					
U5	*Systemic*					
U6	*Legal Urgency*					
	Subtotal					
	TOTAL SCORE					

© not to be copied or reproduced without permission.

Preadmission Triage: Weekly updates of urgency items

Date ___________________________ **Rater** ______________________________

Date ___________________________ **Rater** ______________________________

	**DUNDRUM-2: TRIAGE URGENCY ITEMS**	**0**	**1**	**2**	**3**	**4**
**U 1**	Current Location					
**U2**	Mental Health					
**U3**	Suicide Prevention					
**U4**	Humanitarian					
**U5**	Systemic					
**U6**	Legal Urgency					
	**Subtotal**					

© not to be copied or reproduced without permission.

Date ___________________________ **Rater** ______________________________

Date ___________________________ **Rater** ______________________________

	**DUNDRUM-2: TRIAGE URGENCY ITEMS**	**0**	**1**	**2**	**3**	**4**
**U1**	Current Location					
**U2**	Mental Health					
**U3**	Suicide Prevention					
**U4**	Humanitarian					
**U5**	Systemic					
**U6**	Legal Urgency					
	**Subtotal**					

© not to be copied or reproduced without permission.

DUNDRUM-3 and DUNDRUM-4 six monthly up-dates

Date ___________________________ **Rater** ______________________________

Location

Note that while adding the scores up is not recommended for use as a structured professional judgement instrument, the visual pattern of predominantly 4s and 3s, or 1s and 2s, may help to guide the decision maker regarding readiness for onward movement. Individual item ratings however are the most important guide to treatment planning.

The self-rated DUNDRUM-3 and DUNDRUM-4 may be used side by side with these clinician ratings as a guide to therapeutic concordance.

		**SCORE**				
**DUNDRUM-3:PROGRAMME COMPLETION ITEMS**		**0**	**1**	**2**	**3**	**4**
**PC1**	Physical health					
**PC2**	Mental health					
**PC3**	Drugs and Alcohol					
**PC4**	Problem behaviours					
**PC5**	Self-care and activities of daily living					
**PC6**	Education, Occupation and Creativity					
**PC7**	Family and Social Networks					
	**Subtotal**					
	**DUNDRUM-4: RECOVERY ITEMS**	**0**	**1**	**2**	**3**	**4**
**R1**	Stability					
**R2**	Insight					
**R3**	Rapport and Working Alliance					
**R4**	Leave					
**R5**	HCR-20 Dynamic Items					
**R6**	Victim Sensitivities					
	**Subtotal**					

© not to be copied or reproduced without permission.
